# Vapor‐Assisted Mechanochemical Synthesis of Enzyme and Hydrogen‐Bonded Organic Framework Biocomposites

**DOI:** 10.1002/smll.202504744

**Published:** 2025-06-25

**Authors:** Michael R. Hafner, Natalija Pantalon Juraj, Kate Flint, Helmar Wiltsche, Heimo Wolinski, Heinz Amenitsch, Christian J. Doonan, Krunoslav Užarević, Francesco Carraro

**Affiliations:** ^1^ Institute of Physical and Theoretical Chemistry Graz University of Technology Stremayrgasse 9 Graz 8010 Austria; ^2^ Ruđer Bošković Institute Bijenička c. 54 Zagreb 10000 Croatia; ^3^ Department of Chemistry and Centre for Advanced Nanomaterials University of Adelaide Adelaide SA 5005 Australia; ^4^ Institute of Analytical Chemistry and Food Chemistry Graz University of Technology Stremayrgasse 9 Graz 8010 Austria; ^5^ Institute of Molecular Biosciences, Field of Excellence BioHealth University of Graz Graz 8010 Austria; ^6^ Institute of Inorganic Chemistry Graz University of Technology Stremayrgasse 9 Graz 8010 Austria

**Keywords:** biocomposite, biocatalysis, hydrogen‐bonded organic frameworks, mechanochemistry, vapor‐assisted

## Abstract

Hydrogen‐bonded Organic Frameworks (HOFs) emerged as a matrix for preparing highly active and stable enzyme biocomposites. Conventional biocompatible synthetic procedures in solutions, however, suffer from issues related to competition with the solvent molecules and inhomogeneous loading of the enzyme. Here, it is demonstrated that a combination of mechanochemistry and accelerated aging can be used to synthesize Hydrogen‐bonded Organic Framework (HOF) biocomposites with improved enzyme loading, activity, and protection. Advanced characterization techniques, including in situ Wide‐Angle X‐ray Scattering and Transmission Electron Microscopy, provide insights into these biocomposites' formation mechanisms and structural properties. A comparative analysis with biocomposites prepared via conventional solution synthesis reveals that vapor‐induced growth enhances protein loading, ensures a more homogeneous enzyme distribution, and improves protective properties due to distinct growth mechanisms and kinetics. This simple and green synthetic approach offers a viable alternative to innovative HOF‐based composite materials.

## Graphical abstract

It is demonstrated that a combination of mechanochemistry and accelerated aging can be used to synthesize Hydrogen‐bonded Organic Framework (HOF) biocomposites. The vapor‐induced growth enhances enzyme loading, ensures a more homogeneous enzyme distribution, and improves protective properties due to distinct growth mechanisms and kinetics. This simple and green synthetic approach offers a viable alternative to innovative HOF‐based composite materials.

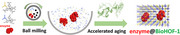

## 1 Introduction

Hydrogen‐bonded Organic Frameworks (HOFs) are a class of porous crystalline materials comprised of organic or metal‐organic building blocks (i.e., tectons) linked together via hydrogen‐bonding interactions. These directional and modular intermolecular bonding motifs can be augmented by additional weak intermolecular interactions (for instance, π−π stacking), and they primarily determine the chemical and physical properties of these materials.^[^ ^1^, ^2^, ^3^, ^4^ ^]^ Although HOFs have been studied for decades, they have recently come into the spotlight due to their high permanent porosity, water stability, and solution processability.^[^ ^5^ ^]^ An emerging area of HOF research is the application of HOFs for the immobilization of enzymes.^[^ ^6^ ^]^ In the last decade, the immobilization of enzymes in self‐assembled porous materials has been attracting increasing attention.^[^ ^7^, ^8^, ^9^, ^10^, ^11^, ^12^ ^]^ In 2019, we reported the first example of an HOF‐based biocomposite, termed BioHOF‐1.^[^ ^13^, ^14^ ^]^ BioHOF‐1 is a microporous HOF synthesized in biocompatible conditions (i.e., water, room temperature) from **1·Cl_4_** = methanetetrayltetrakis(benzene‐4,1‐diyl))tetrakis(aminomethaniminium) and **H_4_2** = 4,4′,4′″,4′″‐methanetetrayltetrabenzoate building units. Following this work, numerous HOF biocomposites have been reported, including derivatives of BioHOF‐1 and even single‐component frameworks such as pyrene‐based HOFs.^[^ ^15^, ^16^, ^17^, ^18^, ^19^, ^20^, ^21^ ^]^ HOF biocomposites demonstrate promising performance in terms of enzyme loading capacity, recyclability, stable operational conditions over a broad pH range, and enhanced protection against chemical/physical stressors.^[^ ^13^, ^16^ ^]^ These properties have spurred interest in the application of enzyme@HOF systems to biocatalysis and biosensing.^[^ ^6^, ^22^ ^]^ Despite these developments, the routes toward HOF biocomposites have, thus far, been limited to one‐pot syntheses in aqueous or organic solvent mixtures.^[^ ^23^ ^]^ In the solution phase, the rapid self‐assembly of HOF components affords limited control over crystal size, morphology, and spatial localization of the enzyme, which are typically located near the crystal surface, affording non‐homogeneous materials.^[^ ^13^, ^20^, ^24^, ^25^ ^]^ Thus, innovative synthetic protocols are required to overcome these limitations and expand the practical scope of HOF biocomposites. In this context, mechanochemical processing,^[^ ^26^, ^27^, ^28^, ^29^, ^30^, ^31^, ^32^, ^33^, ^34^ ^]^ a rapidly growing and scalable solvent‐free synthetic approach that enabled robust pathways towards unconventional materials (including novel HOFs),^[^ ^35^, ^36^, ^37^ ^]^ can play a transformative role in the synthesis of HOF‐based biocomposites.

Here, we show that mechanochemistry combined with accelerated aging strategies^[^ ^38^ ^]^ can be utilized for synthesizing highly active enzyme@M‐BioHOF‐1 composites (where M‐ identifies samples prepared via mechanochemistry). We systematically explore synthetic parameters, including precursor ratios and milling conditions, to optimize enzyme loading, activity, and protection within the biocomposite. We elucidated the formation mechanisms and structural properties of the enzyme@M‐BioHOF‐1, finally comparing them and their activity with enzyme@BioHOF‐1 biocomposites produced via conventional one‐pot solution synthesis (enzyme@S‐BioHOF‐1). Compared to solution‐prepared samples, the distinct growth mechanisms and kinetics of the vapor‐induced enzyme@BioHOF‐1 growth resulted in higher protein loading and uniform protein distribution. These contribute to enhancing both the HOF matrix's protective properties and the biocomposite's specific activity. Thus, this work shows that solid‐state methods are a novel and effective approach to the synthesis of HOF biocomposites.

## 2 Results and Discussion

Mechanochemistry is a green approach to chemical synthesis that typically overcomes issues related to conventional solution processing, such as solubility and solvation,^[^ ^39^ ^]^ while at the same time providing a high level of stoichiometric control^[^ ^40^ ^]^ and reaction selectivity.^[^ ^41^, ^42^ ^]^ Accelerated aging steps (i.e., exposure of the sample to a humid atmosphere) are often combined with mechanochemistry to either improve the crystallinity of the products or to induce the crystallization of the precursors mixed via ball milling.^[^ ^38^ ^]^ A wide variety of solid‐state materials have been synthesized using mechanochemical processing, including porous coordination polymers,^[^ ^43^, ^44^, ^45^ ^]^ and their biocomposites^[^ ^46^, ^47^, ^48^ ^]^ (e.g., enzymes successfully encapsulated in a series of metal‐organic frameworks), and recently, HOFs.^[^ ^35^, ^36^ ^]^ Notably, it was shown that HOF composite materials (e.g., Pd@HOF) that exhibited nano‐sized morphology with uniform inorganic nanoparticle distribution, which is typically challenging or unachievable using solution‐based methods, could be prepared via mechanochemistry.^[^ ^36^ ^]^ This example shows that mechanochemistry could overcome some limitations of HOF biocomposites synthesized in solution. For example, our previous works on BioHOF‐1 biocomposites indicated a non‐homogeneous distribution of the enzyme throughout the composite and afforded limited control of crystal size (i.e., typically needle‐like crystals of ≈ 50 µm and squared section of 1×1 µm).^[^ ^13^, ^20^, ^25^ ^]^ However, the mechanochemical synthesis of HOF biocomposites remains unexplored.

**Figure 1:**
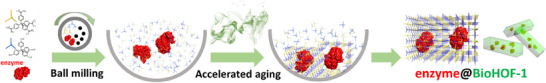
Schematic representation of the mixing of the precursors (1*Cl_4_, H_4_2, and a protein; left) via vibrational dry milling and of the following accelerated aging step, where the crystallization is induced by H_2_O‐NH_3_ vapors (middle), yielding protein@BioHOF‐1 biocomposite with a homogeneous distribution of the protein throughout the crystals (right).

**Figure 2:**
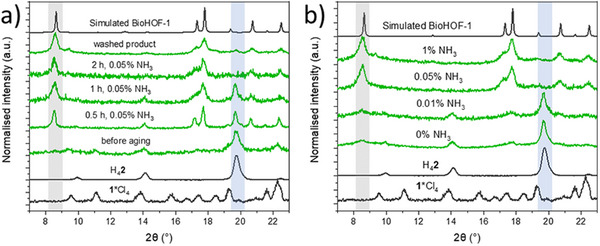
a) XRD patterns of M‐BioHOF‐1 aged for different times in vapors from aqueous 0.05% NH_3_ solutions; b) XRD patterns of M‐BioHOF‐1 aged for 2 h at 4 °C vapors from aqueous NH_3_ solutions of concentrations ranging from 0% to 1%. The growth of the peak of the α‐phase of BioHOF‐1 (8.6°, gray area) and the decrease of the peak of H_4_2 tecton (19.7°, blue area) are highlighted and representative of the conversion of the milled precursors to BioHOF‐1.

In this work, we explore the preparation of BioHOF‐1 composites using solid‐state synthetic techniques and assess how this strategy affects their structure and performance characteristics. A two‐step synthesis approach was employed: the first is dry milling the HOF constituents, followed by accelerated aging of the obtained powder in vapor from a dilute aqueous NH_3_ solution (**Figure** **1**). The dry milling step consists of adding powders of the two BioHOF‐1 building units (**1·Cl_4_** and **H_4_2**) in a jar with ZrO_2_ balls and mixing at 8 Hz. After the dry milling step, to verify if the mechanical energy of the milling process is sufficient to form the BioHOF‐1, we collected the samples and characterized their crystallinity via X‐ray diffraction (XRD). XRD pattern analyses (**Figure** **2**) showed no Bragg maxima associated with any BioHOF‐1 phase^[^ ^49^ ^]^ but matched the sum of the diffraction patterns of the HOF building blocks. To induce crystallization of the HOF without dissolving the building units, we explored a variety of accelerated aging conditions by exposing the dry powders to vapors from aqueous NH_3_ solutions of concentrations ranging from 0% to 1%. We monitored the formation of BioHOF‐1 via XRD and observed that increasing the concentration of ammonia led to faster conversion of the building units to the porous α‐phase of BioHOF‐1 (Figure 2a). For example, using 0.05% NH_3_ solutions or higher, complete conversion of the precursors to BioHOF‐1 was observed within 2 hours (Figure 2b).

However, several hours were needed at lower ammonia concentrations to achieve only partial conversion to BioHOF‐1 (i.e., >2 h, Figure S1, Supporting Information). These data demonstrate that the humid atmosphere of the reactor can promote the molecular mobility of the BioHOF‐1 precursors and facilitate the formation of hydrogen bonds to build the BioHOF‐1 particles. We hypothesize that the faster conversion of precursors to BioHOF‐1 in the presence of NH₃ is due to the deprotonation of carboxylate linkers, leading to a more extensive availability of mobile **H_4_2** molecules for BioHOF‐1 formation. Based on these results, the minimum NH_3_ concentration (i.e., 0.05%) that ensured the complete tectons‐to‐HOF conversion in a relatively short time (i.e., 2 h) was selected for the accelerated aging steps. Following aging, the products were washed with water to remove unreacted precursors. The XRD patterns of the washed samples show that the crystalline phase of the product is unchanged (Figure S1b, Supporting Information).

**Figure 3:**
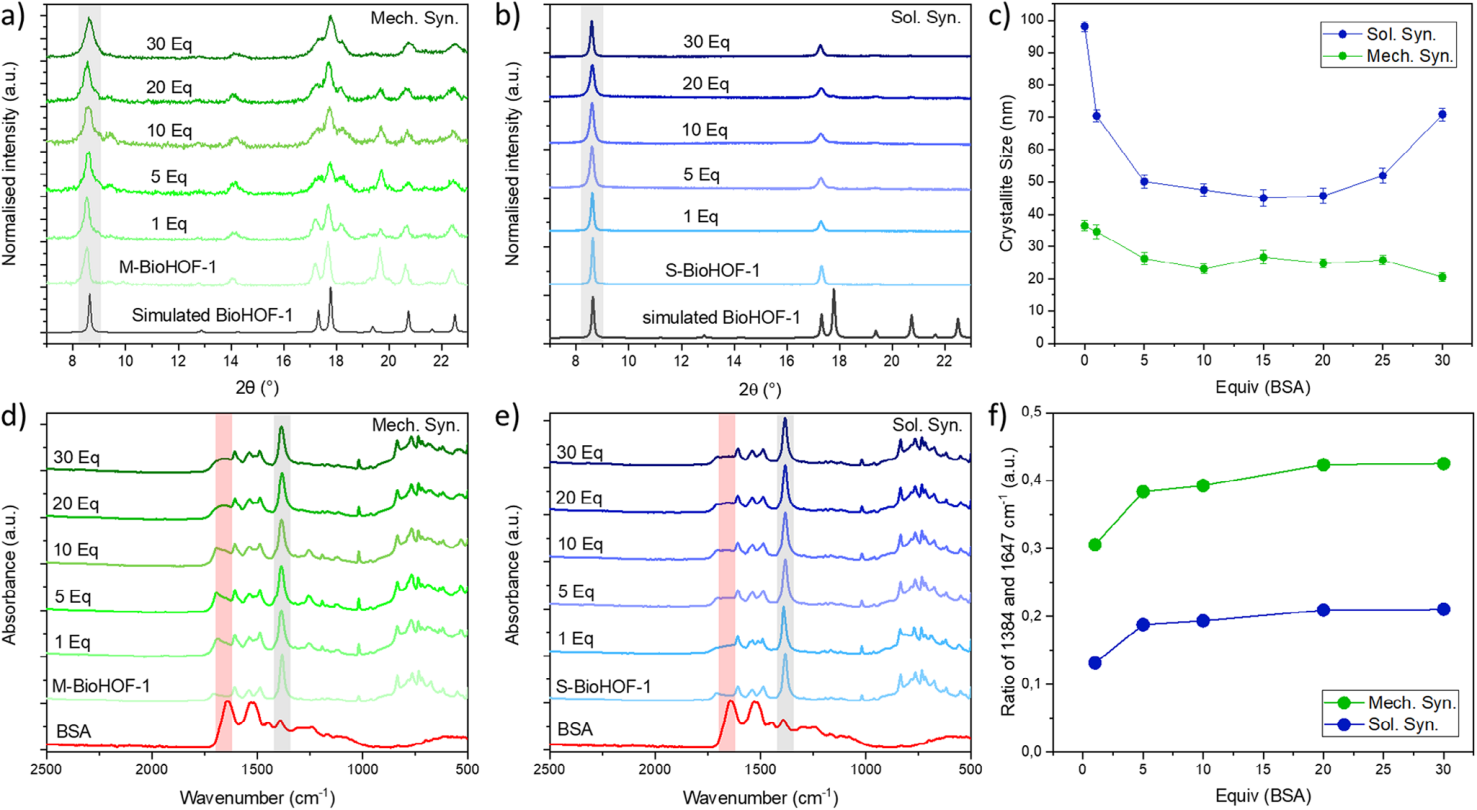
XRD patterns of a) BSA@M‐BioHOF‐1 (Mech. Syn.) and b) BSA@S‐BioHOF‐1 (Sol. Syn.) biocomposites and c) the crystallite size of BioHOF‐1 calculated from the (200) peak of α‐BioHOF‐1 (8.6°, highlighted in gray in a) and b)); ATR‐IR spectra of d) BSA@M‐BioHOF‐1 (Mech. Syn.) and e) BSA@S‐BioHOF‐1 (Sol. Syn.) biocomposites and f) the ratio of the amide I band of BSA (1647 cm^−1^) and the ip ring deform/sym carboxy stretch band of BioHOF‐1 (1384 cm^−1^)^[^ ^51^ ^]^ in the biocomposites.

We then investigated the possibility of using the synthetic protocol optimized for the pure HOF to synthesize protein@M‐BioHOF‐1 biocomposites. Bovine serum albumin (BSA) model protein was selected and introduced as lyophilized powder in the precursor mixture. While the amount of HOF precursors was fixed, an increasing amount of BSA (in the range of 1–30 w/w equivalents of BSA, with 1 eq = 1 mg) was used to prepare a series of BSA@M‐BioHOF‐1 samples. The crystallinity of the samples prepared with BSA was investigated via XRD and revealed that, compared to pure BioHOF‐1, the presence of the protein led to smaller crystallites of the α‐phase^[^ ^49^ ^]^ of BioHOF‐1 and that increasing the amount of protein resulted in decreased crystallite sizes (from 35 nm for 1 eq. BSA@M‐BioHOF‐1 to 20 nm for the 30 eq. BSA@M‐BioHOF‐1; **Figure** **3**a,c). In addition, traces of a partially dehydrated BioHOF‐1 porous phase (i.e., β‐phase, peak at 9.4°)^[^ ^49^ ^]^ can be detected in samples prepared with more than 5 eq of BSA. The presence of the protein in the material was verified via infrared (IR) spectroscopy. The amide I and II bands (1647 cm^−1^ and 1530 cm^−1^)^[^ ^50^ ^]^ in the IR spectra confirmed the immobilization of the protein in the BSA@M‐BioHOF‐1 biocomposites.^[^ ^25^ ^]^ By monitoring the absorbance of the amide I band in the samples prepared with increasing amounts of BSA, we observed an initial increase in the amount of immobilized BSA, followed by a plateau for the samples prepared using ≥10 eq BSA (Figure 3d,f; Figure S2, Supporting Information). The amount of BSA in the final biocomposites was quantified via inductively coupled plasma optical emission spectrometry (ICP‐OES). The data showed comparable loading of protein in the samples prepared using ≥10 eq BSA (maximum protein loading: 15 wt%; Table S1, Supporting Information), supporting the findings from IR spectroscopy studies.

**Figure 4:**
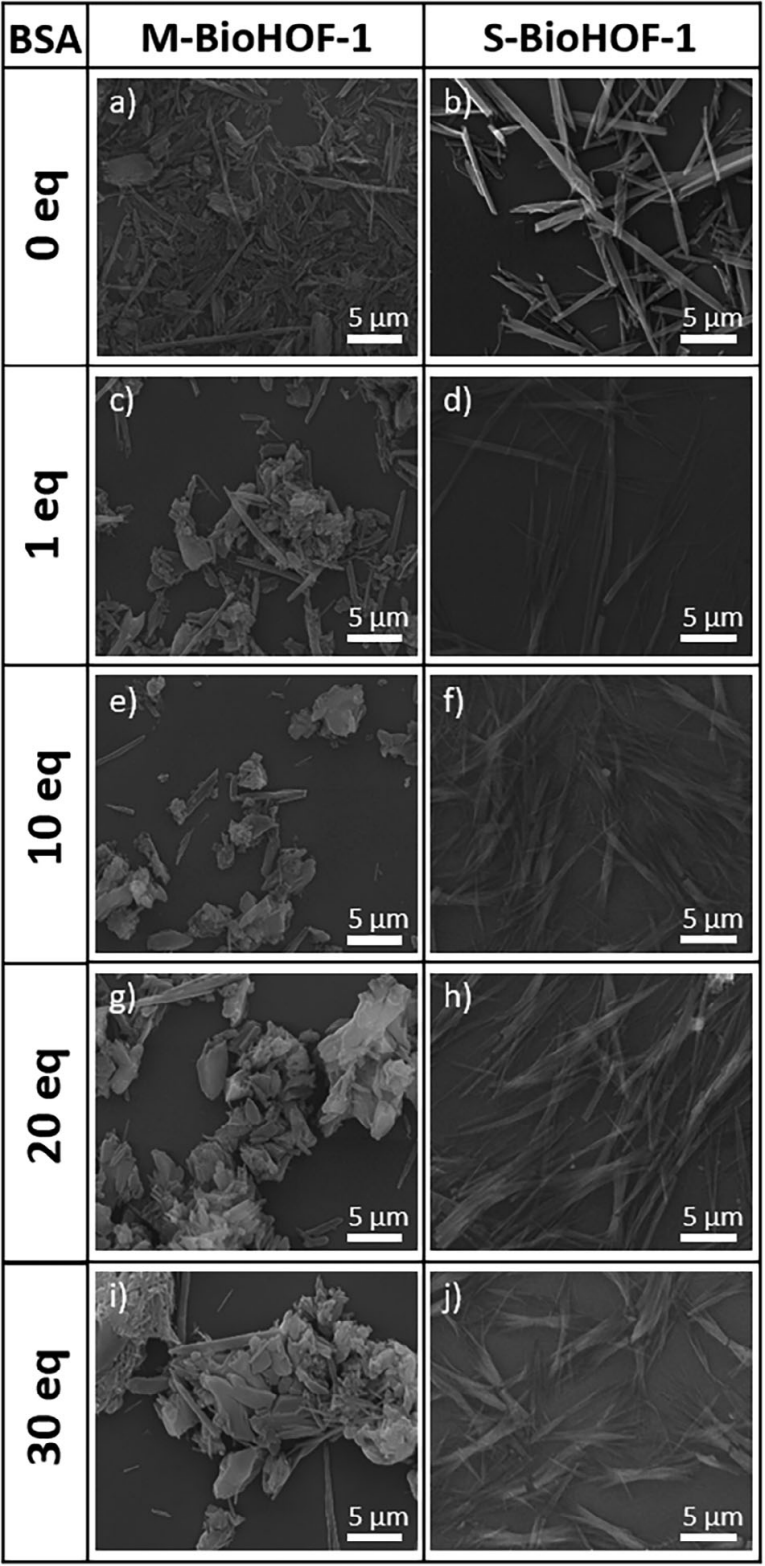
SEM micrographs of a) 0 eq. BSA, M‐BioHOF‐1; b) 0 eq. BSA, S‐BioHOF‐1; c) 1 eq. BSA@M‐BioHOF‐1; d) 1 eq. BSA@S‐BioHOF‐1; e) 10 eq. BSA@M‐BioHOF‐1; f) 10 eq. BSA@S‐BioHOF‐1; g) 20 eq. BSA@M‐BioHOF‐1; h) 20 eq. BSA@S‐BioHOF‐1; i) 30 eq. BSA@M‐BioHOF‐1; j) 30 eq. BSA@S‐BioHOF‐1.

Next, we wanted to assess the impact of the solid‐state synthesis on the morphology of the HOF crystals. The morphology of the M‐BioHOF‐1 and BSA@M‐BioHOF‐1 crystals was investigated via scanning electron microscopy (SEM). SEM micrographs of M‐BioHOF‐1 show the presence of thin rod‐like particles (average size: 0.5 µm × 5 µm) (**Figure** **4**). In the presence of BSA, rod‐like particles of similar width but shorter average length, as well as the presence of particles without a defined morphology in the size range of 1 µm × 1 µm to 5 µm × 5 µm, seemingly composed of aggregated crystals, were observed. The presence of these aggregates appears to dominate with increasing equivalents of BSA, showing a strong influence of the protein/tecton ratio on the morphology of the final biocomposite. It should be noted that the size of the particles/aggregates observed via SEM (Figure 4) is one order of magnitude larger than the average crystallite size calculated from the XRD patterns (Figure 3c). This demonstrates that the biocomposite particles are polycrystalline particles composed of several different crystalline domains.

We then sought to compare the crystallinity, protein loading, and morphology of the biocomposites prepared via solid‐state synthesis (M‐BioHOF‐1) with materials prepared in solution (hereafter, S‐BioHOF‐1). We prepared and characterized S‐BioHOF‐1 via one‐pot solution protocols using the same precursors/protein ratios employed to synthesize M‐BioHOF‐1. The crystallinity of the samples was investigated via XRD, which showed that all samples prepared in solution comprised α‐BioHOF1 crystals (Figure 3b). The analysis of the full width at half maximum (FWHM) of the (200) diffraction peak (Figure 3c) indicates that the samples prepared in solution possess larger crystallites compared to the samples prepared via solid‐state synthesis (i.e., BSA@S‐BioHOF‐1 crystallite size ranges between 70 and 50 nm, while for BSA@M‐BioHOF‐1 it ranges between 35 and 20 nm). Immobilization of BSA in the biocomposites prepared in solution was investigated via IR spectroscopy. The IR spectra of the BSA@S‐BioHOF‐1 samples show both the amide I and amide II bands, confirming the presence of BSA (Figure 3e). To investigate the loading of BSA, the absorbance of the amide I band versus the initial amount of BSA was calculated and plotted (Figure 3f), showing an increase of immobilized BSA in the biocomposite that plateau for the samples prepared using ≥10 eq BSA. When comparing the ratios of the IR bands of the BioHOF‐1 and the amide I band of the immobilized BSA, we can conclude that the samples prepared via solid‐state synthesis have a higher BSA content (Figure 3f). To investigate the differences imparted to the particles' morphology from the different synthetic protocols, we compared the SEM micrographs of the mechanochemically synthesized BSA@M‐BioHOF‐1 samples to the BSA@S‐BioHOF‐1 particles (Figure 4). BSA@S‐BioHOF‐1 particles maintain the predominantly rod‐like shape of the BioHOF‐1 crystals prepared without protein in solution (i.e., ca 1 µm x 10 µm). By increasing the initial BSA content, we observed that the rods have similar lengths but get thinner (ca 0.5 µm) and start aggregating, forming bundles. Overall, we can conclude that the influence of BSA on particle morphology is limited in the BSA@S‐BioHOF‐1 compared to the mechanochemically synthesized BSA@M‐BioHOF‐1.

**Figure 5:**
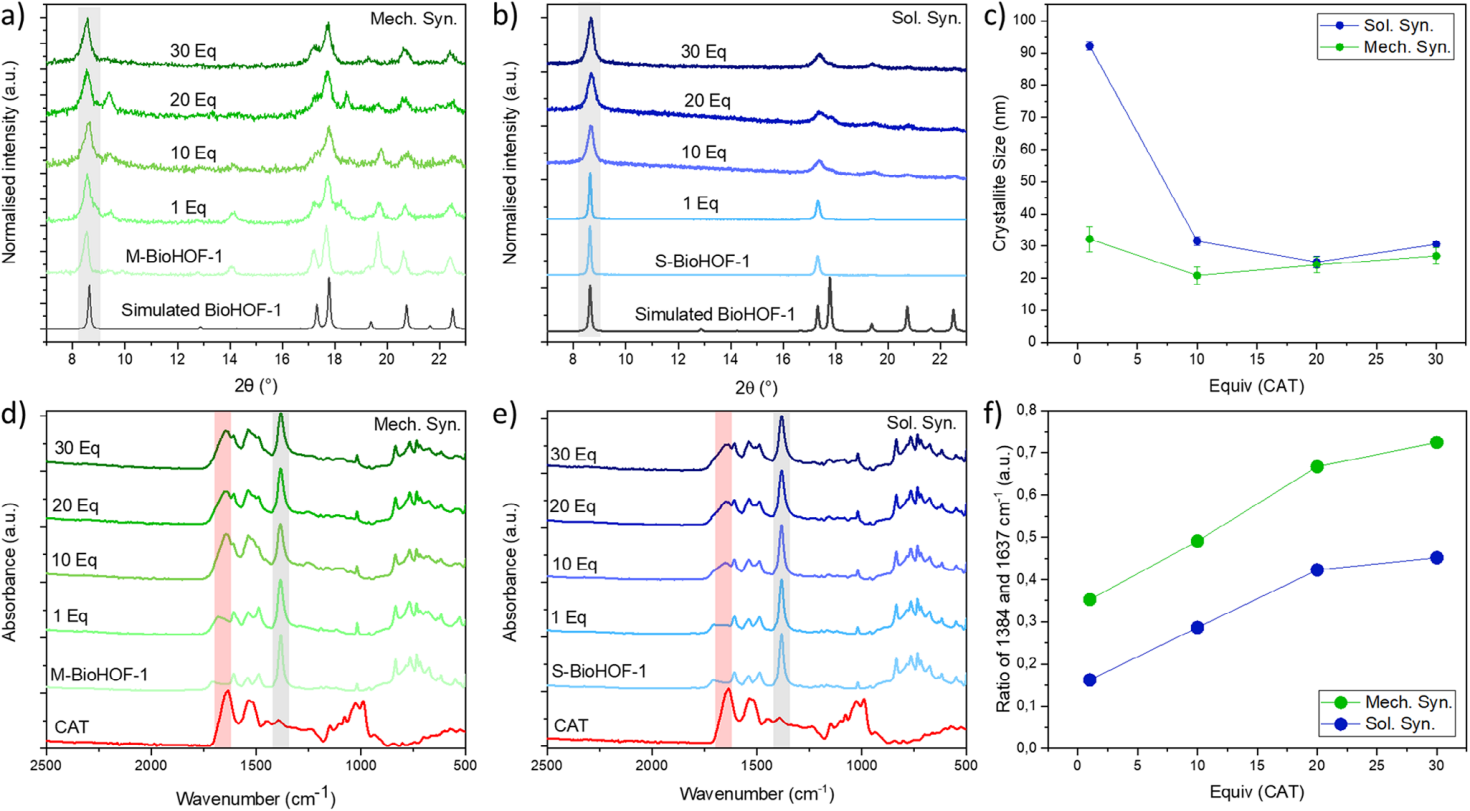
XRD patterns of a) CAT@M‐BioHOF‐1 (Mech. Syn.) and b) CAT@S‐BioHOF‐1 (Sol. Syn.) biocomposites and c) the crystallite size of BioHOF‐1 calculated from the (200) peak of α‐BioHOF‐1 (8.6°, highlighted in gray in a) and b)); ATR‐IR spectra of d) CAT@M‐BioHOF‐1 (Mech. Syn.) and e) CAT@S‐BioHOF‐1 (Sol. Syn.) biocomposites and f) the ratio of the amide I band of CAT (1637 cm^−1^) and the ip ring deform/sym carboxy stretch band of BioHOF‐1 (1384 cm^−1^)^[^ ^51^ ^]^ in the biocomposites.

Next, we investigated the immobilization of an active enzyme in the BioHOF‐1 using the protocol developed for BSA@M‐BioHOF‐1. Catalase (CAT) was selected as the model enzyme for this study. CAT is an oxidoreductase enzyme that catalyzes the decomposition of hydrogen peroxide to water and oxygen, and its enzymatic activity can be analyzed by quantifying the consumption of H_2_O_2_.^[^ ^52^ ^]^ We prepared a series of CAT@M‐BioHOF‐1 samples with increasing enzyme content (i.e., 1, 10, 20, 30 eq) and compared their performance to the biocomposites prepared in solution (CAT@S‐BioHOF‐1).XRD analysis of the CAT@M‐BioHOF‐1 samples showed the formation of the BioHOF‐1 α‐phase (**Figure** **5**a) and crystallite sizes (Figure 5c) similar to those observed for BSA@M‐BioHOF‐1. In addition, traces of a partially dehydrated BioHOF‐1 porous phase (i.e., β‐phase, peak at 9.4°)^[^ ^49^ ^]^ were detected in samples prepared with 1–20 eq. of CAT. IR spectroscopy confirmed the presence of CAT in the samples. By monitoring the intensity of the amide I band (1637 cm^−1^, Figure 5d; Figure S3, Supporting Information) versus the initial amount of CAT, we observed an increase in immobilized CAT, with a plateau at 20 eq. of CAT (Figure 5f). The immobilized CAT was quantified via ICP‐OES, and data showed that the protein loading plateaued at around 55 wt% for CAT@M‐BioHOF‐1 samples prepared with 20 and 30 eq. of CAT, with a maximum loading of 58 wt% for the 20 eq. CAT@M‐BioHOF‐1 sample. This trend of an initial increase in protein loading followed by a plateau is the same as observed for BSA@M‐BioHOF‐1 samples. However, the protein loading is higher in samples containing CAT compared to those containing BSA (i.e., maximum BSA loading: 15 wt%; Tables S1 and S2, Supporting Information): this suggests that the nature of the protein can affect the protein loading of the biocomposite.

SEM micrographs of the 1 eq. CAT@M‐BioHOF‐1 sample show the presence of small rod‐like particles (ca 0.5 µm × 2–5 µm) that tend to aggregate upon drying (Figure S4, Supporting Information). In CAT@M‐BioHOF‐1 samples with higher amounts of CAT micrometric elongated particles are present, with an average size that decreases from 3 µm for the 10 eq. CAT@M‐BioHOF‐1 sample to 0.5−1 µm for the 30 eq.CAT@M‐BioHOF‐1 sample. In some cases (in particular, the 10 eq. CAT@M‐BioHOF‐1 sample), it is still possible to identify the presence of rod‐like particles that aggregate to form the micrometric particles. Conversely, in the case of the 30 eq. CAT@M‐BioHOF‐1 sample, it is not possible to identify any rod‐like particles, and the morphology of the particles is irregular.

We then prepared and characterized the same materials via one‐pot solution protocols (CAT@S‐BioHOF‐1), forming α‐BioHOF1 crystals. The analysis of the FWHM of the (200) diffraction peak shows that the 1 eq. CAT@S‐BioHOF‐1 sample has a larger crystallite size (i.e., 90 ± 1 nm), and increasing initial amounts of CAT result in smaller crystallites (i.e., ranging between 20 and 30 nm) (Figure 5b,c). When monitoring the increase of the amide I band via IR spectroscopy in the CAT@S‐BioHOF‐1 samples, a trend similar to that observed for CAT@M‐BioHOF‐1 biocomposites was detected (Figure 5e,f): the intensity of the amide I band of CAT increases steeply from 1 to 20 eq. CAT and then tends to plateau for 20 and 30 eq. CAT. However, the normalized amide I intensity is lower in the CAT@S‐BioHOF‐1 samples compared to CAT@M‐BioHOF‐1 samples, suggesting a lower loading of CAT in the samples prepared in solution. The loading of CAT in the samples was quantified via ICP‐OES, and the trends observed via IR spectroscopy were confirmed (Table S2, Supporting Information). SEM micrographs show that the 1 eq. CAT@S‐BioHOF‐1 sample comprises large rod‐like crystals (i.e., 1 × 10−20 µm, similar to the bare S‐BioHOF‐1 crystals, Figure S4, Supporting Information). For CAT@S‐BioHOF‐1 samples with higher equivalents of CAT, bundles of smaller rod‐like particles (i.e., 0.5 × 5 µm) are formed. These particles appear to aggregate as the initial amount of CAT is increased. Compared to the BSA@S‐BioHOF‐1 sample, the length of the rods is reduced, and the particles show higher levels of aggregation. This confirms that while the relative concentration of protein and HOF precursors affects the final morphology of the biocomposite, the nature of the protein can also play a key role.

**Figure 6:**
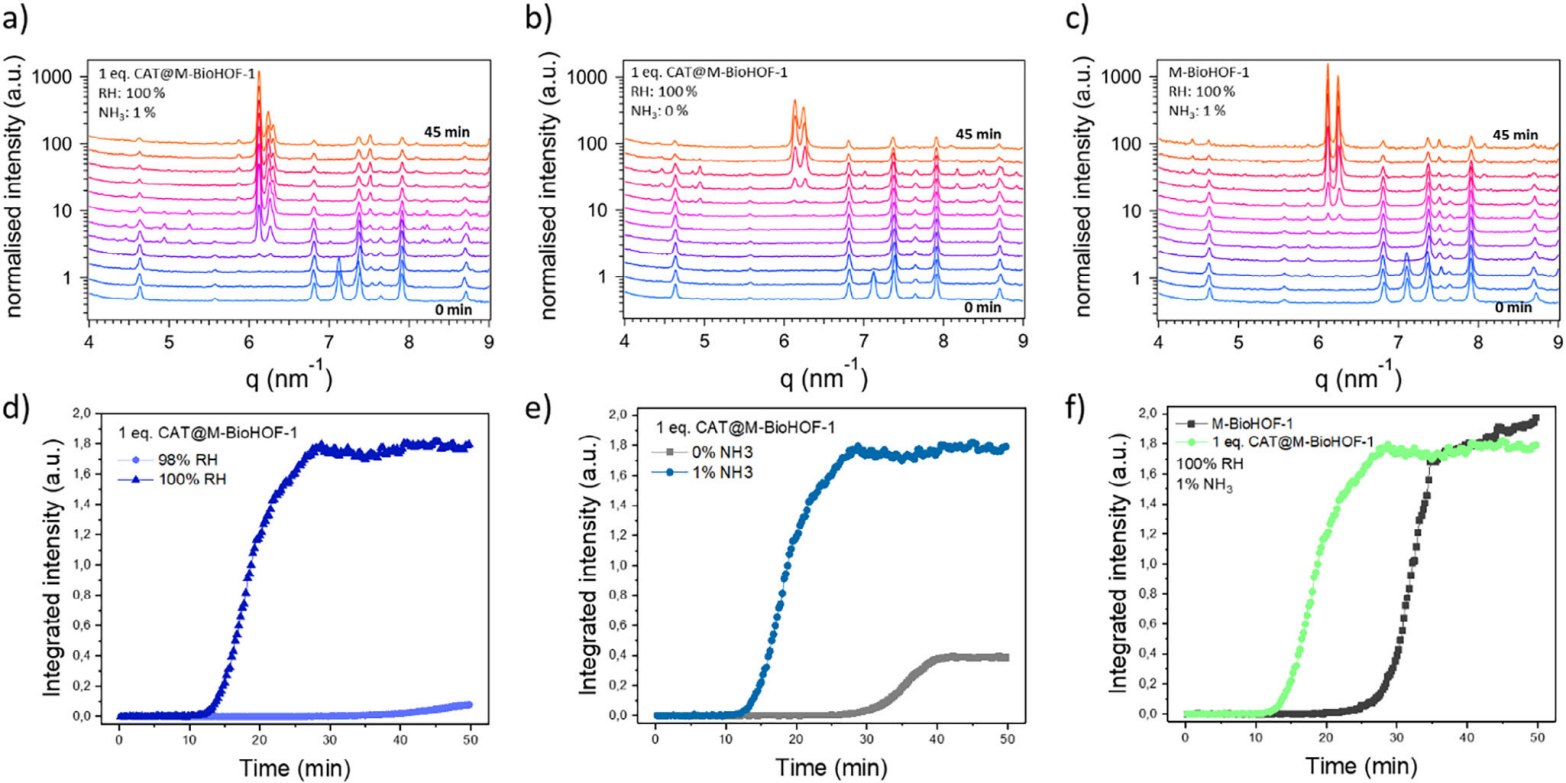
WAXS patterns measured during 45 minutes of in‐flow accelerated aging (note: to enhance readability, the graph displays one pattern every four minutes) of a) 1 eq. CAT@M‐BioHOF‐1 (RH = 100%, NH_3_ = 1%); b) 1 eq. CAT@M‐BioHOF‐1 (RH = 100%, NH_3_ = 0%); c) M‐BioHOF‐1 (RH = 100%, NH_3_ = 1%). Time evolution of the normalized intensity integrated over the 6‐6.45 nm^−1^ scattering vector (q) range (corresponding to the region of the (200) peak of BioHOF‐1) of d) 1 eq. CAT@M‐BioHOF‐1 aged in flow with NH_3_ = 1% and different RHs; e) 1 eq. CAT@M‐BioHOF‐1 aged in flow with RH = 100% and different NH_3_%; f) M‐BioHOF‐1 and 1 eq. CAT@M‐BioHOF‐1 aged in flow (RH = 100%, NH_3_ = 1%).

To gain insights into the mechanism and kinetics of formation for BioHOF‐1 biocomposites via mechanochemistry and accelerated aging, as well as to investigate the role of the protein in the vapor‐assisted crystallization process, we focused on the time‐resolved structural characterization of M‐BioHOF‐1 and CAT@M‐BioHOF‐1. Previous studies have shown that in solution, the kinetics of nucleation of BioHOF‐1 and parent HOFs are not strongly influenced by the presence of the protein.^[^ ^13^ ^]^ In solution, the nucleation of HOF particles is immediate, and the crystallization proceeds within seconds from mixing the precursors.^[^ ^13^ ^]^ To investigate the initial steps of the formation and the crystallization process of M‐BioHOF‐1 and CAT@M‐BioHOF‐1 upon accelerated aging in H_2_O‐NH_3_ vapors, we employed a vapor‐flow‐through setup and in situ synchrotron Wide Angle X‐ray Scattering (WAXS) measurements. A schematic of the in situ experimental setup is shown in Figure S5 (Supporting Information). Using this setup, the influence of CAT on the crystallization of BioHOF‐1 was explored, as well as two parameters of the accelerated aging step that could affect the BioHOF‐1 crystallization: i) the relative humidity (RH) and ii) the NH_3_ concentration (**Figure** **6**; Figures S6–S14, Supporting Information). First, we investigated the synthesis of M‐BioHOF‐1. In the absence of the protein, after 9 minutes of exposure to a flow of H_2_O‐NH_3_ vapors, we observed a rapid decrease in the intensity of the diffraction peaks of **H_4_2** (e.g., 7.1 nm^−1^(10°), Figure 6a; Figure S6, Supporting Information). Within 2 minutes, the diffraction peaks corresponding to **H_4_2** were not detectable anymore, suggesting the loss of crystallinity of the carboxylate tecton. However, at this stage, significant changes were not observed in the peaks corresponding to the amidinium tecton (**1·Cl_4_**). We ascribed this to the sufficient amount of solvent molecules to dissolve the carboxylate tecton and allow for mobility.^[^ ^53^ ^]^ The basicity of the environment and the limited amount of solvent favor the dissolution of the carboxylate tecton over the chlorine salt of the amidinium. After 25 minutes, we detected the presence of the (200) peak of the α‐phase of BioHOF‐1 (6.1 nm^−1^ (8.6°)), as well as a second peak at 6.4 nm^−1^ (9°). We hypothesize that this second peak could be ascribed to a phase of BioHOF‐1 with an intermediate degree of hydration between the fully hydrated α‐phase and the partially dehydrated β‐phase.^[^ ^49^ ^]^ As the reaction proceeded, the intensity of both these peaks increased rapidly until a plateau was reached after 35 minutes (Figure 6a,d). In parallel, the intensity of the amidinium tecton peaks decreased as the amidinium tecton reacted slowly with the dissolved carboxylate to yield BioHOF‐1 crystals. In the presence of protein (i.e., 1 eq. CAT), after 6 minutes we observed a rapid decrease in the intensity of the diffraction peaks of **H_4_2,** and after 12.5 minutes, we observed the appearance of the BioHOF‐1 diffraction peaks (Figure 6b,d; Figure S7, Supporting Information). The intensity of the BioHOF‐1 peaks grew rapidly, seeming to plateau after 25 minutes of reaction. Compared to the pure HOF synthesized under the same conditions (Figure 6d), we observe that the presence of the protein accelerates the i) loss of crystallinity of the carboxylate tecton (6 vs 9 minutes), ii) first appearance of the BioHOF‐1 diffraction peaks (12.5 vs 25 minutes) and iii) time for the crystallization to plateau (25 vs 35 minutes). We attribute this faster BioHOF‐1 formation to the fact that the protein is added in the precursors' mixture as lyophilized powder: the hygroscopic nature of the lyophilized protein facilitates a local increase of water, causing mobility of tecton molecules and, ultimately, the formation of the BioHOF‐1.

In the flow setup, the influence of the concentration of solvent molecules was also investigated by changing the relative humidity of the carrier flow. By reducing the humidity by only 2%, we observed that, in flow, the reaction did not proceed within 2 hours for the pure HOF (Figure S8, Supporting Information). In the presence of CAT, almost 40 minutes were required to detect the appearance of very broad BioHOF‐1 diffraction peaks (Figure 6e; Figure S9, Supporting Information). These experiments illustrate the critical role the protein plays, favoring the accumulation of solvent molecules in the reaction mixture to accelerate the BioHOF‐1 formation.

We also investigated the role of NH_3_ in the BioHOF‐1 crystallization by performing analogous experiments using a 100% RH flow without any NH_3_ (Figure 6c,f; Figures S10, S11, Supporting Information). We observed that, in the absence of protein, no BioHOF‐1 was formed within 130 minutes (Figure 6c,f; Figure S10, Supporting Information). These data confirm the hypothesis that the presence of NH_3_ facilitates the deprotonation of the carboxylate tectons, allowing faster accumulation of dissolved **H_4_2** molecules and leading to BioHOF‐1 formation.

The same experiment was performed in the presence of CAT to verify if the protein could facilitate the BioHOF‐1 crystallization in the absence of NH_3_. In the presence of CAT, we observed the disappearance of the **H_4_2** peaks in the same time frame measured in the presence of NH_3_ (i.e., 6–8 minutes, Figure 6c; Figure S11, Supporting Information). We then detected the growth of the BioHOF‐1 diffraction peaks after 30 minutes of reaction, with the integrated intensity plateauing after 40 minutes (Figure 6c,f; Figure S11, Supporting Information). These times are longer than those measured in the presence of both NH_3_ and 1 eq. CAT (i.e., 12.5 and 25 minutes, respectively), showing that the crystallization of CAT@M‐BioHOF‐1 is slower in the absence of NH_3_. However, BioHOF‐1 formation is still observed, demonstrating that the presence of the protein can favor the local increase of water molecules and enable the crystallization of BioHOF‐1 despite the absence of NH_3_.

**Figure 7:**
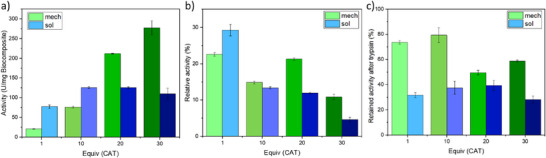
Summary of the catalase enzymatic assay results of CAT@M‐BioHOF‐1 and CAT@S‐BioHOF‐1 biocomposites: a) specific activity of the biocomposites; b) relative activity of the immobilized CAT (%, compared to free CAT); c) relative enzymatic activity of the biocomposites after trypsin digestion (%, compared to the activity of the sample prior trypsin digestion).

We then evaluated the enzymatic activity of CAT immobilized in CAT@M‐BioHOF‐1 biocomposites, and we compared it to the free CAT and the CAT@S‐BioHOF‐1 biocomposites (**Figure** **7**a,b; Figures S15–S17, Supporting Information). The enzymatic assay quantifies the kinetics of H_2_O_2_ consumption in potassium phosphate buffer (50 mM, pH 7.1) by directly measuring the H_2_O_2_ concentration over time. By comparing the enzymatic activity of free CAT with the performance of the biocomposites, we noticed that the retained activity upon immobilization of CAT ranges between 25% and 15% for the CAT@M‐BioHOF‐1 samples (Figure 7b). Analyzing the specific activity of the biocomposites (i.e., activity per mass of biocomposite, U/mg, Figure 7a), we observe that, in the case of CAT@M‐BioHOF‐1 samples, the specific activity increases with the increase of the initial amount of CAT, reaching a maximum of 280 U/mg in the case of 30 eq. CAT@M‐BioHOF‐1. It should be noticed that, despite showing similar CAT loadings by ICP‐OES, the 30 eq. CAT@M‐BioHOF‐1 shows higher specific activity than the 20eq samples: this is most likely due to the smaller particle size of the 30eq sample and a more efficient substrate diffusion through the particles. These data suggest that the specific activity of the CAT@M‐BioHOF‐1 can be effectively increased by increasing the starting mass of CAT and that the particle size could play a role in the performance of the biocomposites prepared via mechanochemistry.

Conversely, the retained activity of the CAT@S‐BioHOF‐1 samples decreases from 30% (1 eq. CAT@S‐BioHOF‐1) to 5% (30 eq. CAT@S‐BioHOF‐1) as the initial CAT concentration increases. Analyzing the specific activity, we observe that the CAT@S‐BioHOF‐1 samples show an increase from 75 to 120 U/mg when the initial CAT is increased from 1 to 10 eq. Then, despite the increase in initial CAT and the loading of CAT in the final material (e.g., the CAT content increases from 38 wt% to 55 wt% from 10 eq. CAT@S‐BioHOF‐1 to 20 eq. CAT@S‐BioHOF‐1), the specific activity remains stable around 120 U/mg. These data demonstrate that in the case of CAT@S‐BioHOF‐1, an increase in CAT loading in the final material does not necessarily lead to an increase in the activity of the biocomposite. Furthermore, these data show that the maximum activity observed for the samples prepared via solid‐state synthesis (i.e., 280 U/mg) is 2.3 times higher than the one measured for the samples prepared via solution synthesis (i.e., 120 U/mg), suggesting that solid‐state synthesis could be applied to prepare highly active materials.

The recyclability of the CAT@M‐BioHOF‐1 samples was investigated and compared with the recyclability of the CAT@S‐BioHOF‐1 samples and the free CAT. To test the recyclability, we performed 10 consecutive enzymatic assays on each sample and calculated the retained activity over the number of cycles. Free CAT loses 50% of the activity after 3 cycles and is completely deactivated after 5 cycles (Figure S18, Supporting Information). The loss of activity of free CAT is attributed in the literature to the irreversible binding of H_2_O_2_ to the active site of CAT, and this limits the amount of substrate that each enzyme can process effectively.^[^ ^54^, ^55^ ^]^ On the other hand, CAT@M‐BioHOF‐1 samples showed a significant increase in activity, retaining, on average, 40% activity after 10 cycles. Among these samples, the 10eq sample is the best‐performing one, with >95% retained activity in the first 4 cycles and around 75% after 8 cycles, Figure S18 (Supporting Information). In the case of CAT@S‐BioHOF‐1 samples, the activity of CAT is partially preserved in the first 3 cycles, with an average retained activity of 60% (Figure S19, Supporting Information). After these initial cycles, the retained activity further decreases and plateaus at around 10% after 10 cycles. The recyclability test results demonstrate that CAT is stabilized within M‐BioHOF‐1 compared to free CAT and that the encapsulated enzyme can process up to 3 times higher amounts of H_2_O_2_ before losing 50% of its activity (i.e., Figures S18, S20, Supporting Information).

We hypothesized that the differences in enzymatic activity for samples prepared via mechanochemistry and those prepared in solution could result from a different spatial localization of CAT within the HOF particles. Therefore, we examined the spatial localization of the protein in the HOF particles. To distinguish between surface‐exposed and fully encapsulated CAT, we followed a trypsin digestion protocol to inactivate any surface‐exposed CAT (Figure 7c).^[^ ^20^ ^]^ Then, we measured the enzymatic activity of the trypsin‐treated samples and compared the performance with the untreated samples. This allowed us to calculate the percentage of retained activity and quantify the presence of surface‐exposed CAT. In the literature, this approach showed how trypsin could access most of the enzyme immobilized in BioHOF‐1 synthesized in solution.^[^ ^20^, ^25^ ^]^ First, we tested the samples prepared via solid‐state synthesis. The 1 eq. CAT@M‐BioHOF‐1 sample retained ca 75% of the initial activity, the 10 eq. CAT@M‐BioHOF‐1 retained ca 80% of activity, and the 20 and 30 eq. samples retained 50% and 60% of activity, respectively. These data suggest that, for samples prepared via the mechanochemical approach (CAT@M‐BioHOF‐1), most of the enzyme is not accessible to trypsin, and therefore, it is fully encapsulated in the HOF. Conversely, for the samples prepared via the solution approach (CAT@S‐BioHOF‐1), only 30% of the original enzymatic activity was retained after trypsin digestion for the 1 eq. CAT@S‐BioHOF‐1 sample, with 40% retained activity for both the 10 and 20 eq. CAT samples and 30% retained activity for the 30 eq. CAT@S‐BioHOF‐1 sample. The data suggest that the amount of surface‐exposed CAT varies in the CAT@S‐BioHOF‐1 samples from 70 to 60%. These results demonstrate that CAT@M‐BioHOF‐1 samples showed a retained activity after exposure to trypsin significantly higher compared to the CAT@S‐BioHOF‐1, and this could be ascribed to a higher percentage of fully encapsulated enzyme in the samples prepared via solid‐state synthesis.

**Figure 8:**
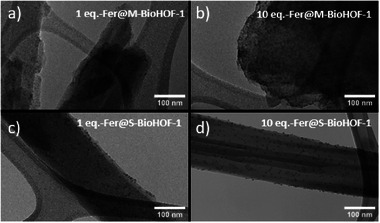
TEM micrographs of a) 1 eq. Ferritin@M‐BioHOF‐1, b) 10 eq. Ferritin@M‐BioHOF‐1, c) 1 eq. Ferritin@S‐BioHOF‐1, d) 10 eq. Ferritin@S‐BioHOF‐1.

To investigate the localization of the enzyme in the biocomposites, we prepared a set of samples using fluorescein isothiocyanate‐tagged CAT (FCAT), and characterized them via confocal laser scanning microscopy (CLSM). We synthesized a series of FCAT@M‐BioHOF‐1 samples and, for comparison, FCAT adsorbed on pre‐synthesized CAT@M‐BioHOF‐1 biocomposites (FCAT‐on‐CAT@M‐BioHOF‐1). The CLSM images (Figures S21, S22, Supporting Information) show a difference between the samples with FCAT immobilized in the particles and with FCAT adsorbed on pre‐synthesized biocomposites. In the case of the FCAT@M‐BioHOF‐1 samples, the fluorescent signal is homogeneously distributed through the particles. On the other hand, when FCAT is adsorbed on CAT@M‐BioHOF‐1 particles, the images show a clear localized emission from the particles’ surfaces. The data suggest that the FCAT is encapsulated within the M‐BioHOF‐1 particles, and these results align with the trypsin digestion results. To further investigate the protein localization in the HOF crystals, we prepared a series of biocomposites using ferritin as a probe protein (Figure S23, Table S3, Supporting Information). Ferritin (Fer) is an iron‐storage intracellular protein with high Fe content (i.e., up to 25 wt%) that can be exploited for imaging purposes: transmission electron microscopy (TEM) can be used to image the localization of ferritin embedded in matrixes with lower electronic contrast.^[^ ^56^ ^]^ In the case of the BioHOF‐1, the organic nature of the crystals allows for high contrast between the iron of the ferritin and the HOF matrix. TEM micrographs of the Fer@M‐BioHOF‐1 samples show that ferritin is homogeneously distributed within the HOF crystals and that the density of ferritin per crystal is higher in the 10, 20, and 30 eq. samples compared to the 1eq‐Fer@M‐BioHOF‐1 sample (**Figure** **8**a,b; Figures S24–S27, Supporting Information). In the case of the Fer@S‐BioHOF‐1 samples, TEM micrographs show that ferritin is mostly localized close to the surface of the crystals in all the investigated samples, with a minor increase in the density of ferritin per crystal observed for the samples prepared with higher Fer concentration, (Figure 8c,d; Figures S28–S32, Supporting Information). The differences in the protein distribution can be related to the different mechanisms of biocomposites formation. Using in situ WAXS, it was found that crystallization is accelerated by the presence of the protein in the homogeneous precursors' mixture prepared by vibrational milling, suggesting the protein can act as a heterogeneous nucleating agent. Conversely, in the one‐pot solution synthesis, the presence or absence of the protein did not affect the HOF formation kinetics.^[^ ^7^ ^]^ Furthermore, compared to the rapid synthesis in solution, the slower kinetics of BioHOF‐1 formation and the limited mobility of the precursors during the vapor‐assisted crystallization step can favor protein encapsulation throughout the growing BioHOF‐1 particles.

Collectively, the results of the in situ WAXS, the trypsin‐digested samples, and the CLSM and TEM investigations show that the combination of the dry milling and accelerated aging steps led to BioHOF‐1 biocomposites with a homogeneous spatial distribution of protein within the crystals.

## 3 Conclusion

The presented work demonstrates a novel synthetic strategy for the rapid and efficient preparation of enzyme@BioHOF‐1 biocomposites. This highly effective synthetic approach results in a homogenous distribution of the enzyme throughout the HOF crystal and facilitates control of enzyme loading. We propose that the homogeneous mixing of the HOF precursors via mechanochemical processing and the slow vapor‐assisted crystallization favor the observed spatial distribution of proteins and lead to improved protein loading capacity compared to samples prepared in solution. As a result, enzyme@M‐BioHOF‐1 biocomposites show superior protection against proteolytic agents and higher specific activity and recyclability than their solution‐prepared counterparts or the free enzyme. This study confirms that this simple and green approach is an effective protocol for synthesizing new, high‐quality, and customizable protein@HOF biocomposites. Accordingly, future works focused on low‐solubility guests or coformers can expand the library of HOF biocomposites, unlocking new potential application areas for these innovative materials.

## Conflict of Interest

The authors declare no conflict of interest.

## Supporting information



Supporting Information

## Data Availability

The data that support the findings of this study are available from the corresponding author upon reasonable request.
