# Supplementary material for: Vapor‐Assisted Mechanochemical Synthesis of Enzyme and Hydrogen‐Bonded Organic Framework Biocomposites
Source: Small. 2025 Jun 25;21(33):2504744. doi: 10.1002/smll.202504744 (PMC12372444; doi:10.1002/smll.202504744)
Supplement: Supplementary file 1 — Supporting Information [file SMLL-21-2504744-s001.pdf]

NANO · MICRO
small

Supporting Information

for *Small*, DOI 10.1002/smll.202504744

Vapor-Assisted Mechanochemical Synthesis of Enzyme and Hydrogen-Bonded Organic Framework Biocomposites

Michael R. Hafner, Natalija Pantalon Juraj, Kate Flint, Helmar Wiltsche, Heimo Wolinski, Heinz Amenitsch, Christian J. Doonan, Krunoslav Užarević and Francesco Carraro**

Vapor-assisted Mechanochemical Synthesis of Enzyme and Hydrogen-bonded Organic Framework Biocomposites.

Michael Hafner[‡,1,a], Natalija Pantalon Juraj[‡,1,b], Kate Flint[c], Helmar Wiltsche[d], Heimo Wolinski[e], Heinz Amenitsch[f], Christian J. Doonan[c], Krunoslav Užarević[b,*], Francesco Carraro[a,*]

[a] Institute of Physical and Theoretical Chemistry, Graz University of Technology, Stremayrgasse 9, Graz, 8010 Austria

[b] Ruđer Bošković Institute, Bijenička c. 54, 10000 Zagreb, Croatia

[c] Department of Chemistry and Centre for Advanced Nanomaterials, University of Adelaide, Adelaide, South Australia, 5005 Australia

[d] Institute of Analytical Chemistry and Food Chemistry, Graz University of Technology, 8010 Graz, Austria

[e] Institute of Molecular Biosciences, Field of Excellence BioHealth, University of Graz, Graz 8010, Austria

[f] Institute of Inorganic Chemistry, Graz University of Technology, 8010 Graz, Austria

Supporting Information

Experimental

General remarks. All chemicals and solvents were purchased from commercial sources and were used as received. X-ray diffraction (XRD) patterns were collected (0.02°/step, 0.3 seconds/step) on a PANalytical Aeris diffractometer (Cu-Kα) at room temperature. IR spectra were measured on a PerkinElmer UATR Two spectrometer.

Characterization. Enzymatic activity assays based on UV-Vis spectroscopy (for Catalase) were performed using a Thermo Scientific NanoDrop One. The concentration of the protein in the was routinely analysed via the BCA assay. FT-IR measurements were done with an alpha Bruker using ATR mode (128 Scans, resolution 4 cm^{-1}). XRD measurements were done using a Rigaku SmartLab X-Ray Diffractometer (9 kW, Cu source, λ= 1.5406 Å). SEM micrographs were collected with a TESCAN Vega (20 kV acceleration voltage, sputter gold coating of samples).

Mechanochemical synthesis of protein@BioHOF-1. The synthesis was carried out under optimized conditions in 6 mL PMMA jars with twenty 0.2 g ZrO_2 balls on an InSolido Technologies IST 500 mixer mill. In a typical procedure, Tetrakis(4-amidiniumphenyl)methane tetra hydrochloride (**1·Cl$_4$**) (4 mg) and enzyme (10 mg = 10 equiv) were milled for 0.5 min at 8 Hz, then Tetrakis(4-carboxylic acid phenyl) methane (**H$_4$2**) (3 mg) was added and the milling continued for another 1 min.

The resulting powder was transferred onto a flat surface (watch glass) to be maximally exposed to the NH_3 vapors, placed in an aging chamber with 0.05% NH_3 and aged for 2 h at 4°C. In samples where a low amount of powder was obtained, the jar was opened and placed in the aging chamber without the transfer step. The aged sample was washed, dispersed, and

centrifuged three times with distilled water to remove any unreacted precursors and loosely adsorbed enzyme.

It should be notice that, in an aqueous solution, BioHOF-1 nucleation is rapid (i.e. HOF nucleation starts within 100 ms from the mixing of the tectons and the crystallization starts within seconds).[7] Therefore, to limit the uncertainty related to small differences in the delay between the introduction of water and the start of the milling process, we envisioned avoiding the solution medium and using dry milling to efficiently and reproducibly mix the precursors.

Solution Synthesis of BioHOF-1. The synthesis of BioHOF-1 was performed according to the literature.[13] 4 mg of **1·Cl$_4$** in 1 ml DI water to form solution A. 3 mg of **H$_4$2** are dispersed in 0.95 ml DI water, followed by the addition of 0.05 ml an aqueous ammonia solution (1% v/v) to form solution B. Solution B is slowly and dropwise added under stirring and at room temperature (21°C) to solution A. The reaction mixture was left stirring for 1 h. The product, a white powder, was obtained through centrifugation and washing three times with DI water. The powder was dried at room temperature and ambient conditions for 48 h.[13]

Solution Synthesis of protein@BioHOF-1. The synthesis of BioHOF-1was performed according to the literature.[13] 4 mg of **1·Cl$_4$** are dissolved in 0.25 ml DI water to form solution A. Depending on which equivalents of enzyme are chosen for the synthesis of the biocomposite, varying amounts of an enzyme stock solution (40 mg/ml) are added. For 1 enzyme equivalent samples 25 µl of the enzyme stock solution plus 725 µl of DI water are added to solution A and stirred gently for 10 min to form solution B. After the 10 min of stirring, solution B should be used immediately. (For the 10 enzyme equivalents samples 250 µl of enzyme stock plus 500 µl DI water are added. For the 20 enzyme equivalents samples 500 µl of enzyme stock plus 250 µl DI water are added. For the 30 enzyme equivalents samples 750 µl of enzyme stock are added.) 3 mg of **H$_4$2** are dispersed in 0.95 ml DI water, followed by the addition of 0.05 ml of an aqueous ammonia solution (1% v/v) to form solution C. Solution C is slowly and dropwise added under stirring and at room temperature (21°C) to solution B. The reaction mixture was left stirring for 1 h. The product, a white powder, was obtained through centrifugation and washing three times with each 1 ml DI water. The powder was dried at room temperature and ambient conditions for 48 h. The samples used for the enzyme activity assays were not dried. Instead these samples were re-dispersed after the washing procedure in each 1 ml DI water and used immediately.[13]

Catalase Assay. The enzymatic activity of catalase and catalase@BioHOF-1 biocomposites was tested using a modified protocol from Sigma Aldrich (EC 1.11.1.6). 100 µl of the suspension of the freshly synthesised and washed catalase@BioHOF-1 biocomposite particles are combined with 900 µl of a potassium phosphate buffer (50 mM, pH 7.1). 967 µl of a H2O2 solution (15 mM) in potassium phosphate buffer (50 mM, pH 7.1) are mixed under gentle stirring at room temperature with 33 µl of either catalase stock solution (0.1 mg/ml in potassium phosphate buffer, 50 mM, pH 7.1) or 33 µl of the catalase@BioHOF-1 biocomposite suspensions. The addition of either catalase or catalase@BioHOF-1 biocomposite starts the reaction. The absorption at 240 nm is recorded (Spectrophotometer: NanoDrop One UV-Vis by Thermo Fisher).

Catalase Recycling Assay. The enzymatic activity of catalase and catalase@BioHOF-1 biocomposites was tested using a modified protocol from Sigma Aldrich (EC 1.11.1.6). 100 µl of the suspension of the freshly synthesised and washed catalase@BioHOF-1 biocomposite particles are combined with 900 µl of a potassium phosphate buffer (50 mM, pH 7.1). 967 µl of a potassium phosphate buffer (50 mM, pH 7.1) are mixed under gentle stirring at room temperature with 33 µl of either catalase stock solution (0.1 mg/ml in potassium phosphate buffer, 50 mM, pH 7.1) or 33 µl of the catalase@BioHOF-1 biocomposite suspensions. The suspension is slightly stirred to prevent sedimentation and dissipate any O_2 bubbles that form during the assay. 1 µL of a 30% H_2O_2 solution in water is added. This accounts for a final concentration of H_2O_2 of 15 mM. The addition of H_2O_2 starts the reaction. The absorption at 240 nm is recorded. After full consumption of H_2O_2, a new cycle is started by the addition of again 1 µL of a 30% H_2O_2 solution. The full consumption of H_2O_2 is reached when the measured absorption at 240 nm reaches the starting value before the addition of H_2O_2. The kinetic of H2O2 consumption is measured and used to calculate the activity of catalase. This procedure is repeated 10 times.

Preparation of Fluorescein isothiocyanate tagged Catalase (FCAT). Fluorescein isothiocyanate (FITC, 0.25 mg) and Catalase (CAT, 20 mg) were dissolved in 2 mL carbonate-bicarbonate aqueous buffer solution (0.1 M, pH 9.2) and stirred gently for 2 h under the exclusion of light at room temperature (21°C). The FCAT was recovered by passing the reaction mixture through an Illustra G-25 column. The crude tagged-enzyme solution was concentrated through a 10 kDa membrane (Microcon®-10, Ultracel® PL-10) by centrifugation (13k rpm for 8 min), followed by solvent-exchange with DI water. The solvent-exchange process was repeated three times to ensure the complete removal of buffer salts in the solution. The purified FCAT was dried in a desiccator under vacuum in the presence of $CaCl_2$. The dried powder was stored under the exclusion of light in the freezer (-20°C).

Confocal laser scanning microscopy (CLSM). Images were acquired using a Leica SP8 DLS confocal microscope with spectral detection (Leica Microsystems, Inc.) and a Leica HC PL APO 63×/1.2 W CORR CS2 objective. Prior to imaging, coverslip correction was performed following the manufacturer's instructions to optimize the fluorescence signal. FITC was excited at 488 nm, and emission was detected between 500 nm and 550 nm. Images were acquired with a scan speed of 700 Hz, a pinhole size of 1 Airy unit, and a sampling density of 42x42 nm (x/y) and measured using a photomultiplier tube. Fluorescence and transmission images were acquired simultaneously.

ICP-OES. The concentration of S was quantified by radially viewed inductively coupled optical emission spectrometry (ICP-OES; Arcos II, Spectro, Germany). Samples were prepared via microwave assisted digestion: 10 – 40 mg sample material were digested with 4 ml HNO_3 (concentrated; purified by subboiling) at 200 °C and 20 bar using a Multiwave GO (Anton Paar, Austria) microwave assisted sample digestion system. Temperature program comprised of a 20 min ramp from room temperature to 200 °C and this temperature was maintained for 10 minutes. After cooling to room temperature, the clear digests were made to a final volume of 20 ml. The analyte quantification was performed using the S (I) 180.731 nm emission line. Scandium was used as internal standard at a concentration of 1 mg L^{-1}.

TEM. TEM micrographs were collected on a Philips CM200 Transmission Electron Microscopy. Samples were dispersed in ethanol and drop-cast onto 3 mm TEM grids prior to analysis.

Proteolytic Agent Treatment. The retained enzymatic activity, upon exposure to a proteolytic agent, of catalase and catalase@BioHOF-1 biocomposites was tested using a modified protocol from Sigma Aldrich. 100 µl of the suspension of the freshly synthesised and washed catalase@Bio-HOF 1 biocomposite particles are combined with 900 µl of a Trypsin solution in potassium phosphate buffer (50 mM, pH 7.1) to yield a final concentration of trypsin of 2 mg/ml. The mixture is left at static conditions at room temperature for 2 h. Afterwards, the suspension is used for measuring the respective retained enzymatic activity. For the free catalase, 100 µl of an aqueous catalase solution is combined with 900 µl of a Trypsin solution in potassium phosphate buffer (50 mM, pH 7.1) to yield a final concentration of trypsin of 2 mg/ml. The final catalase concentration was 0.1 mg/ml. The rest of the procedure was identical to the assay explained in the section "Catalase Assay".

WAXS in-situ crystallization upon humid NH_3 vapor stream. These in-situ experiments have been done at the Austrian SAXS beamline at the Elettra Synchrotron in Trieste (Italy).[57] The synthesis was carried out under optimized conditions in 6 mL PMMA jars with twenty 0.2 g ZrO_2 balls. For the 0 equiv. experiment, **1·Cl$_4$** (12 mg) and **H$_4$2** (9 mg) were milled via shaking by hand at moderate speed for 1 min. For the 1 equiv. experiment, **1·Cl$_4$** (12 mg) and enzyme (3 mg = 1 equiv.) were milled via shaking by hand at moderate speed, then **H$_4$2** (9 mg) was added and the milling continued for another 1 min. A flow-through glass capillary (open on both ends) is carefully packed with the mixed and milled respective powder to yield a dense packing of the powder at the center of the capillary. To avoid any movement of the powder, downstream of the powder, cotton is added and densely packed. A stream of humid air with controlled humidity[58] at relative humidities (RH) of 50%, 98% and 100% and a flow rate of 1 sL/min, which is achieved by mixing dry and 100% humidified air at the desired ratio, is combined with a T-connector with a stream of 10 % NH_3 and a flow rate of 0.1 sL/min. This set-up yields a final NH_3 concentration in the stream of 1 %. The combined streams are then flushed through the capillary, which is packed with the powder and the cotton. The outlet of the stream leads into a fume hood. A schematic of the set-up is given in Figure S15.

The WAXS in-situ measurements of the crystallization were conducted at a photon energy of 8 keV (0.154 nm) and covering a scattering vector q range from 0.16 nm^{-1} until 9.17 nm^{-1}, which accounts for a 2θ range (Cu-source) from 0.23° up to 12.91°. A Pilatus3 1M, Dectris Ltd detector (Baden, Switzerland; sample to detector distance: 750 mm) was used. The exposure time of each collected WAXS pattern was 5 s and the exposure period was 15 s.

The data have been normalized by transmission and corrected by the intensity fluctuations.

Influence of low NH_3 concentration and extended aging time and the washing on the crystallinity of M-BioHOF-1

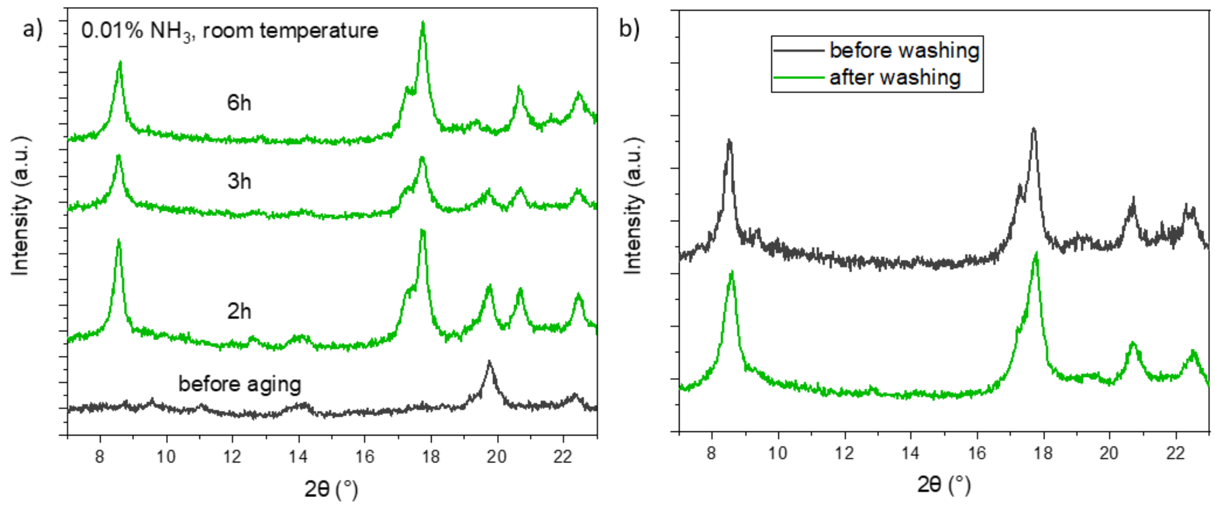

Figure S1: PXRD patterns that shows the a) Influence of a low NH_3 vapour concentration (0.01%) and extended aging time on the crystallinity of M-BioHOF-1, and b) influence of the water washing of the aged powder on the crystallinity of M-BioHOF-1.

Analysis of the Amide I band in BSA@M-BioHOF-1 biocomposites

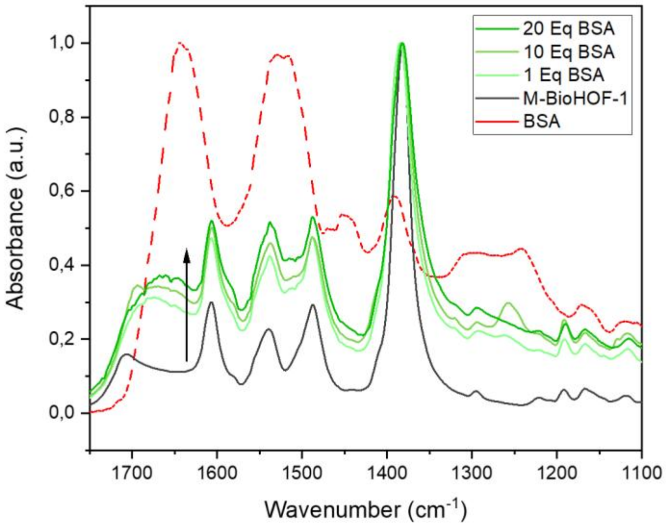

Figure S2: Detail of the IR spectra of BSA, M-BioHOF-1 and 1,10,20 eq. BSA@M-BioHOF-1.

Loading of BSA in BSA@M-BioHOF-1 biocomposites

Table S1: BSA loading (wt%) and encapsulation efficiency (EE%) calculated from ICP-OES.

Mechanochemical Synthesis		
**BSA equivalents**	**wt%**	**EE%**
1	6%	50%
5	9%	20%
10	11%	18%
15	12%	17%
20	11%	15%
25	15%	19%
30	12%	15%

Analysis of the Amide I band in CAT@M-BioHOF-1 biocomposites

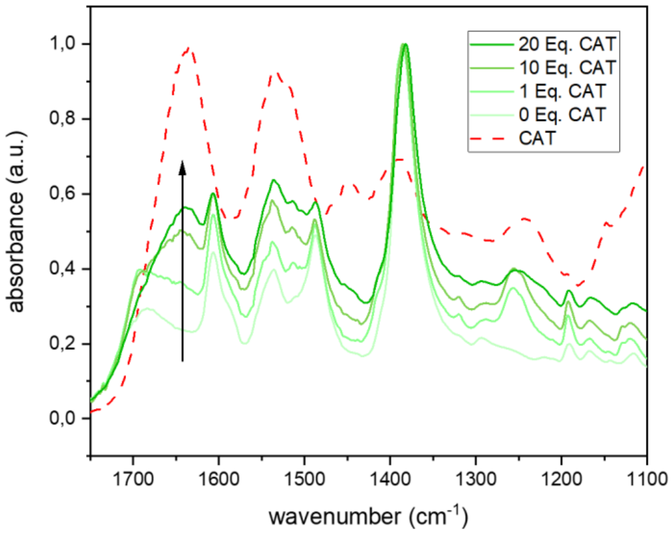

Figure S3: Detail of the IR spectra of CAT, M-BioHOF-1 (i.e. 0 eq.) and 1,10,20 eq. CAT@M-BioHOF-1.

Loading of CAT in CAT@M-BioHOF-1 biocomposites

Table S2: CAT loading (wt%) and encapsulation efficiency (EE%) calculated from ICP-OES.

Mechanochemical Synthesis		
**CAT equivalents**	**wt%**	**EE%**
1	13%	103%
5	24%	58%
10	42%	72%
15	43%	63%
20	58%	78%
25	47%	60%
30	54%	67%
**Solvothermal Synthesis**		
**CAT equivalents**	**wt%**	**EE%**
1	9%	68%
10	36%	61%
20	55%	74%

SEM of CAT@M-BioHOF-1 biocomposites

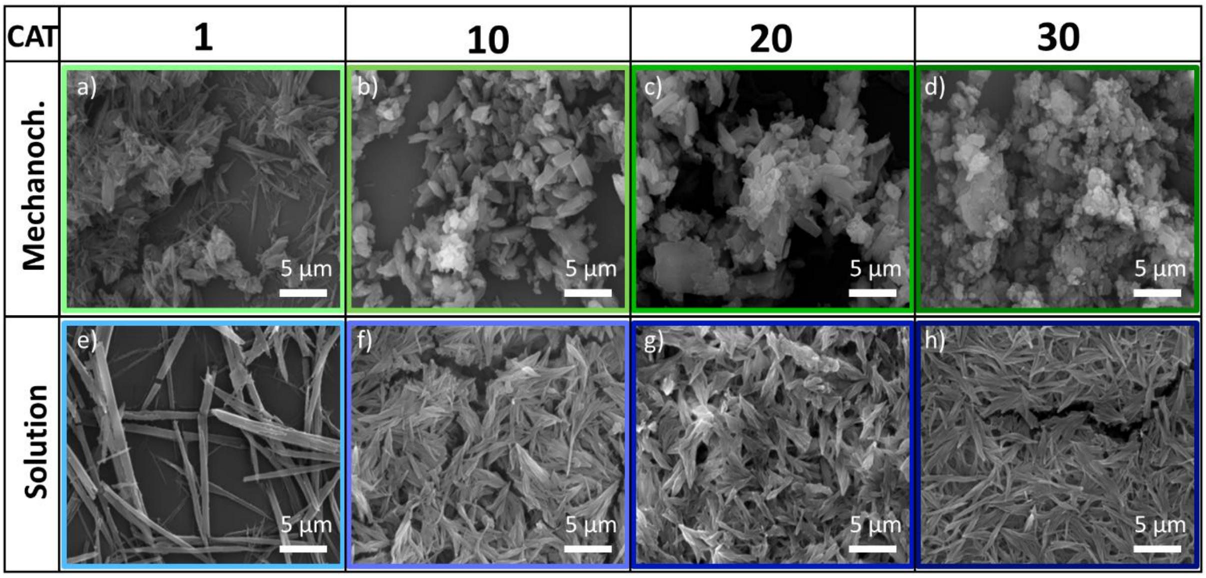

Figure S4: SEM micrographs of CAT@M-BioHOF-1: a) 1 eq. CAT@M-BioHOF-1, b) 10 eq. CAT@M-BioHOF-1, c) 20 eq. CAT@M-BioHOF-1, d) 30 eq. CAT@M-BioHOF-1 and CAT@S-BioHOF-1: e) 1 eq. CAT@S-BioHOF-1, f) 10 eq. CAT@S-BioHOF-1, g) 20 eq. CAT@S-BioHOF-1, h) 30 eq. CAT@S-BioHOF-1

In-situ WAXS measurements with the vapour flow through set-up

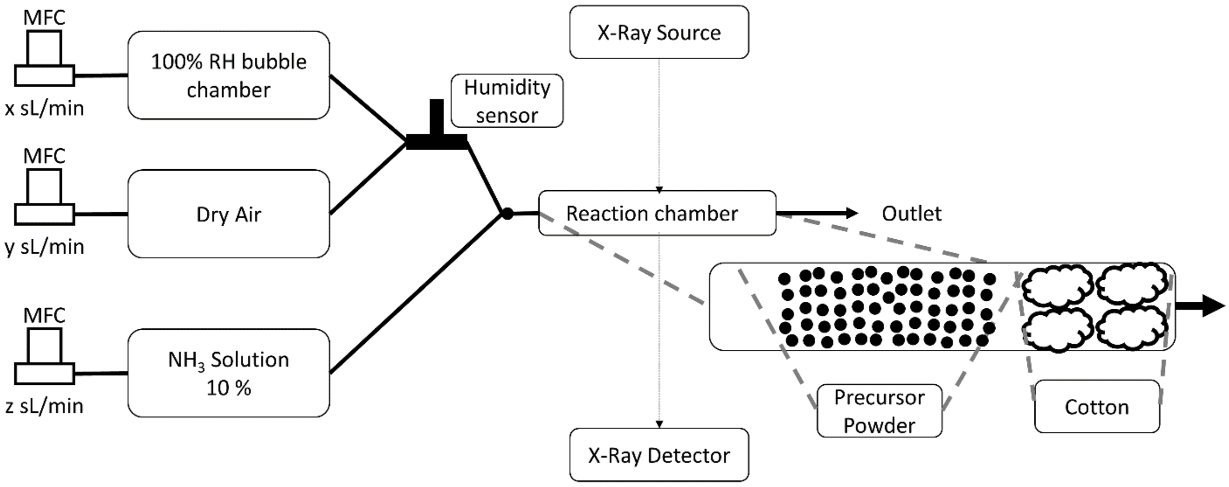

Figure S5: schematic of the set-up for the in-situ WAXS measurements with the vapour flow-through set-up. The flow rate of the MFC of the NH_3 Solution chamber (z) is either 0.1 sL/min (for experiments named "1% NH_3") or 0 sL/min (for experiments named "0% NH_3"). The flow rate of the mass-flow controller (MFC) of the 100% RH bubble chamber (x) plus the flow rate of the MFC of the Dry Air chamber (y; used only when a RH<100% was needed) yield a final flow rate of these combined streams of either 1 sL/min or 1.1 sL/min. If no NH_3 is present (0% NH_3), the sum of x and y is 1.1 sL/min. If NH_3 is present (1% NH_3), the sum of x and y is 1. Therefore, the final flow (x+y+z) is always set at 1.1 sL/min. The NH_3 Solution chamber was at 25°C: taking into consideration that the partial pressure of ammonia for a 10% solution is approximately 0.1 atm[59], we approximated the final NH_3 concentration to 1% by volume and named the experiments accordingly (i.e. "1% NH_3").

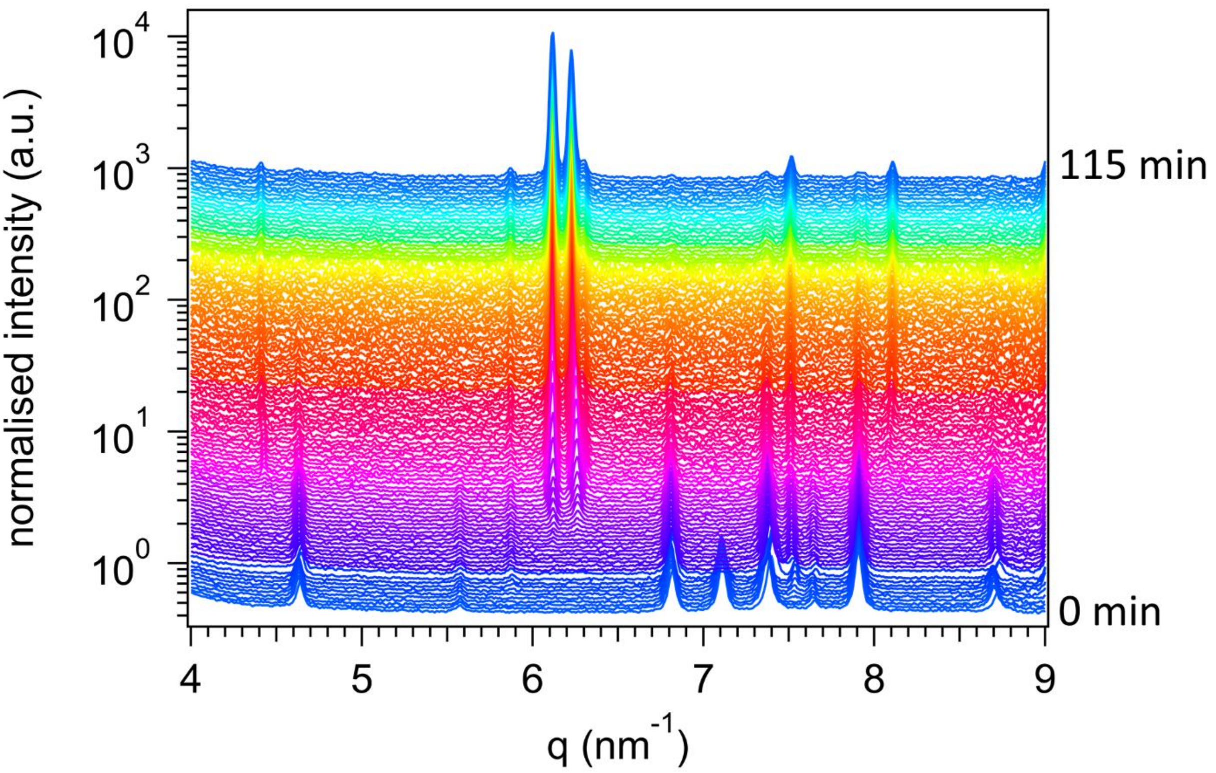

Figure S6: in-situ WAXS measurement of the crystallisation of M-BioHOF-1 with a flow of 1 sL/m of air with 100% RH and a flow of 0,1 of 10% NH_3 vapour, resulting in a NH_3 concentration in the vapour of 1%. The time between each frame is 1 min.

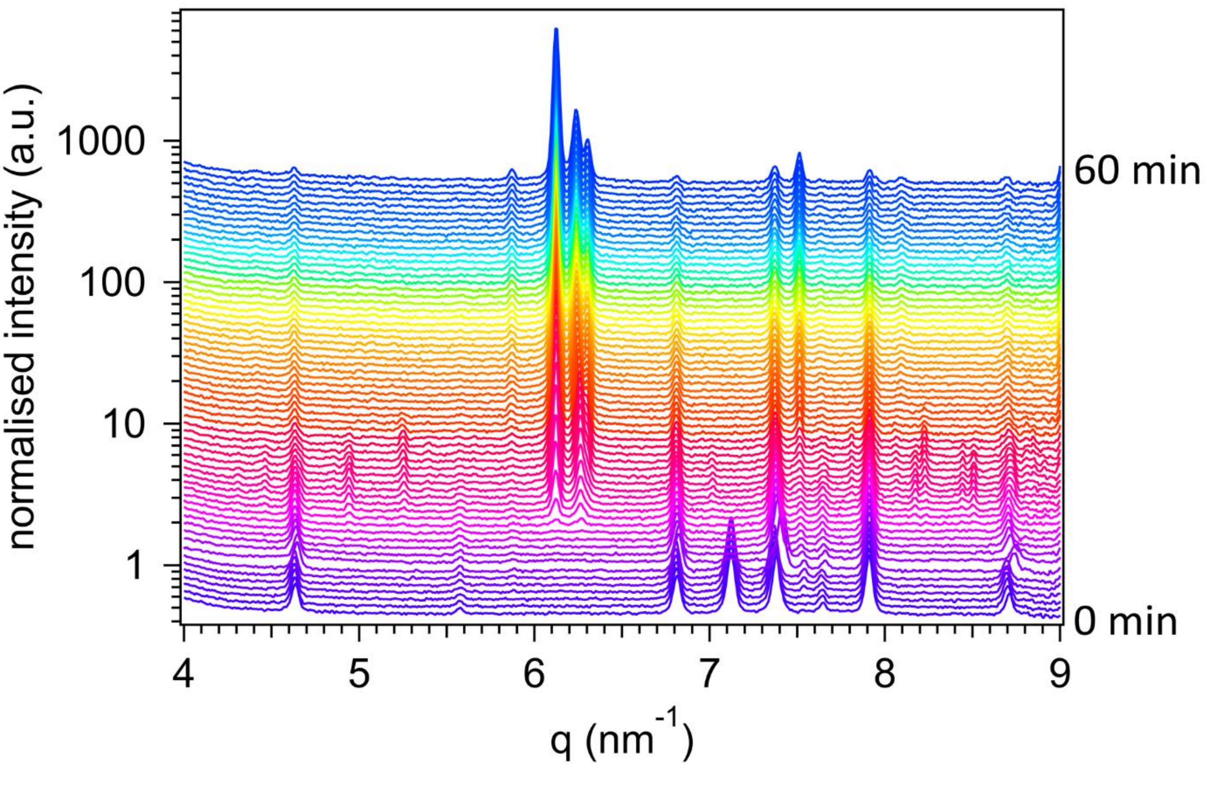

Figure S7: in-situ WAXS measurement of the crystallisation of 1-eq. CAT@M-BioHOF-1 with a flow of 1 sL/m of air with 100% RH and a flow of 0.1sL/M of 10% NH_3 vapour, resulting in a NH_3 concentration in the vapour of 1%. The time between each frame is 1 min.

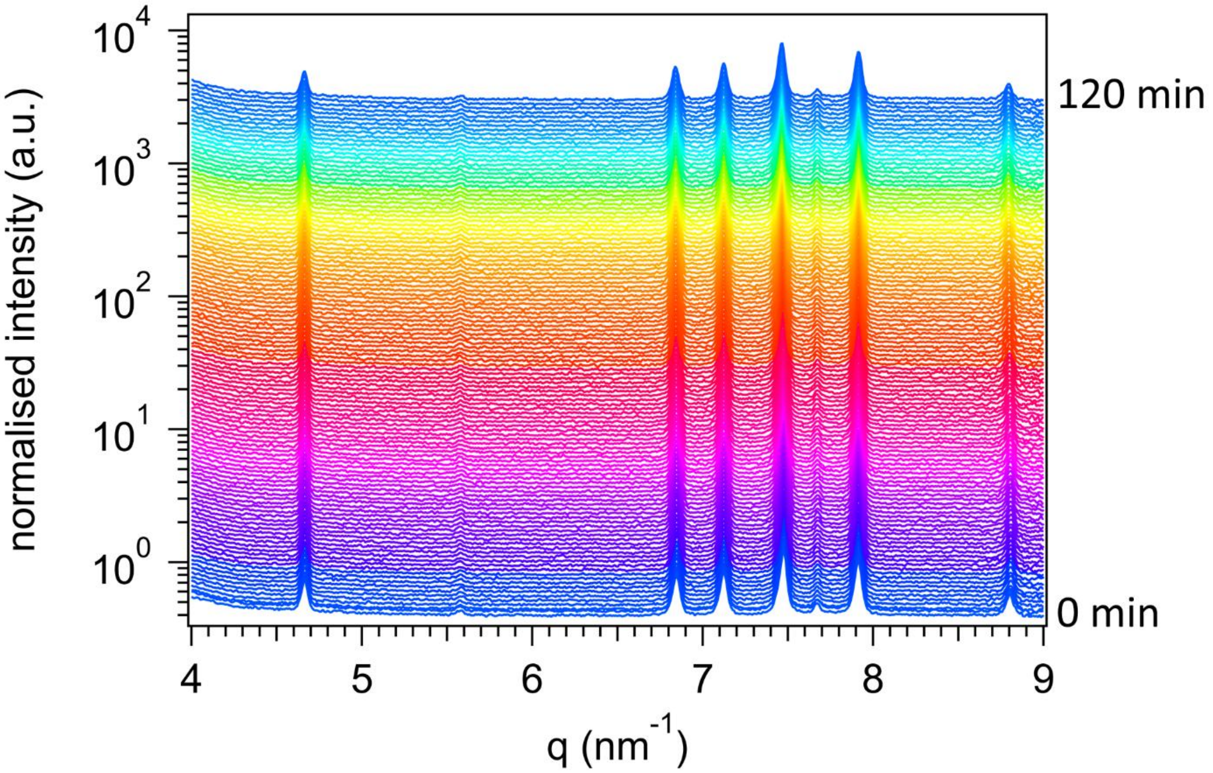

Figure S8: in-situ WAXS measurement of the crystallisation of M-BioHOF-1 with a flow of 1 sL/m of air with 98% RH and a flow of 0,1 of 10% NH_3 vapour, resulting in a NH_3 concentration in the vapour of 1%. The time between each frame is 1 min.

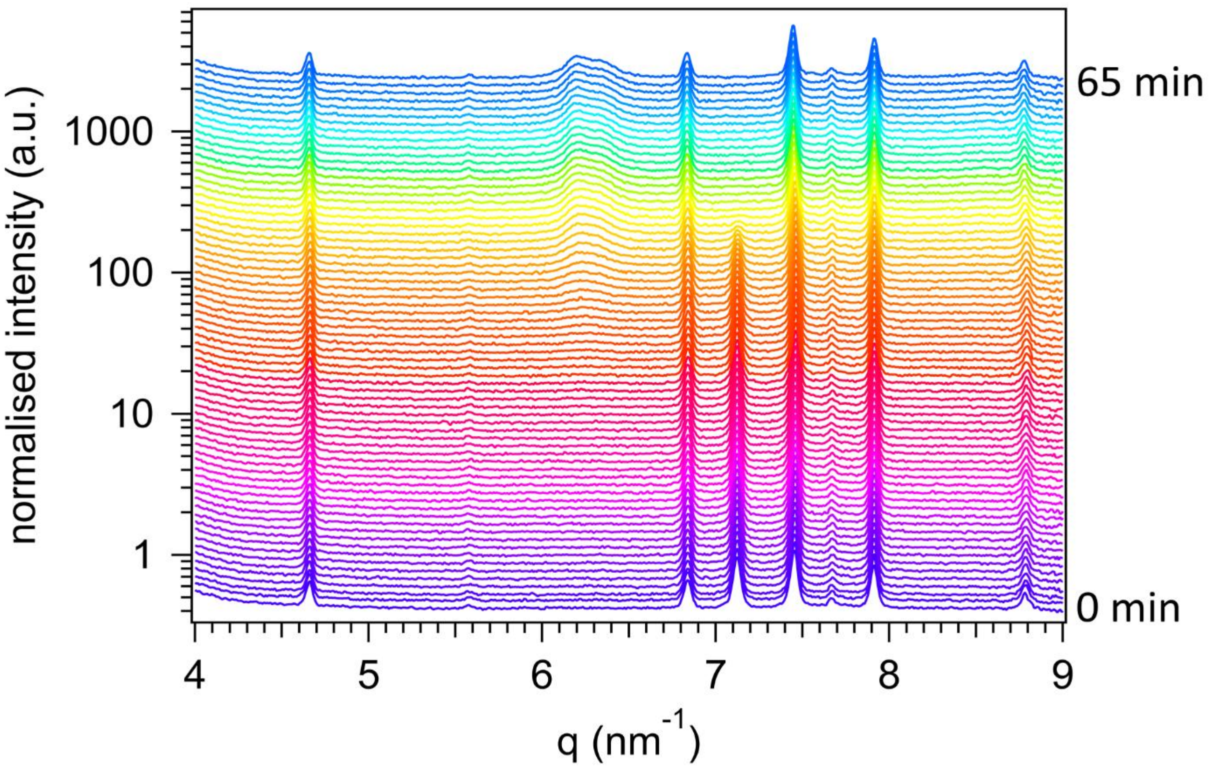

Figure S9: in-situ WAXS measurement of the crystallisation of 1-eq. CAT@M-BioHOF-1 with a flow of 1 sL/m of air with 98% RH and a flow of 0.1 of 10% NH_3 vapour, resulting in a NH_3 concentration in the vapour of 1%. The time between each frame is 1 min.

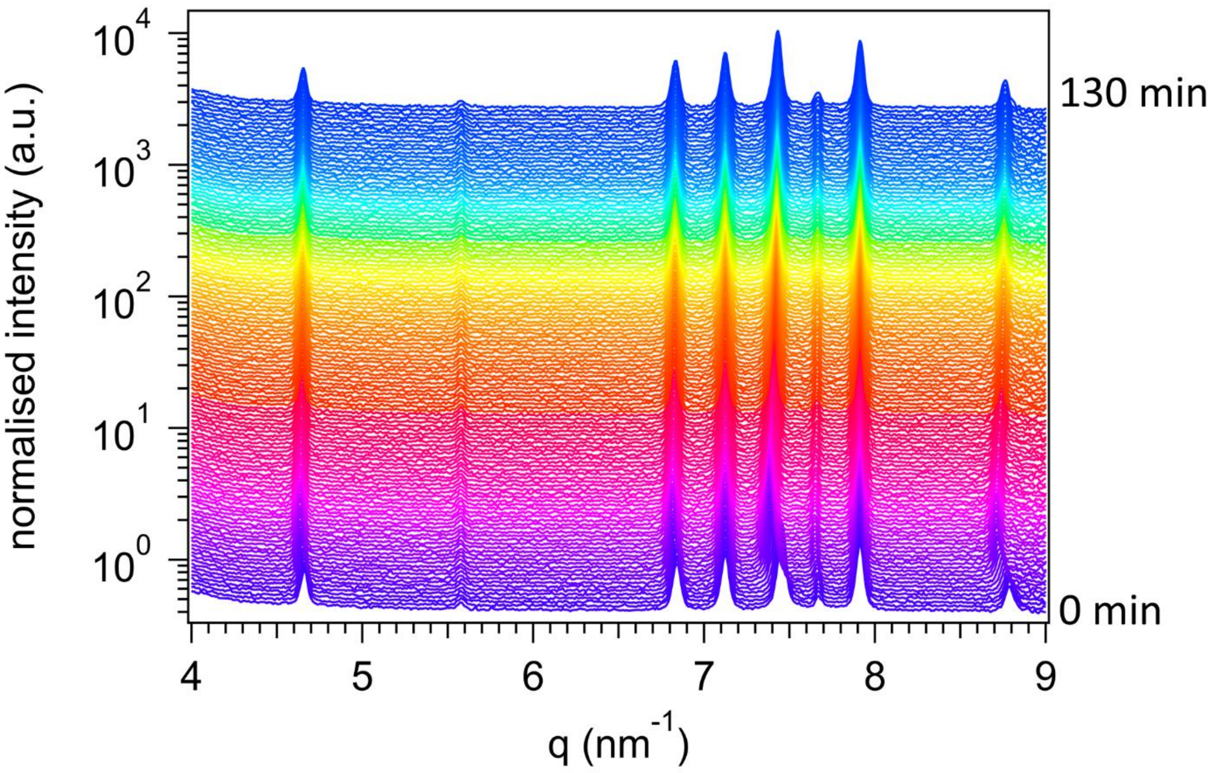

Figure S10: in-situ WAXS measurement of the crystallisation of M-BioHOF-1 with a flow of 1.1 sL/m of air with 100% RH and no flow of NH_3 vapour. The time between each frame is 1 min.

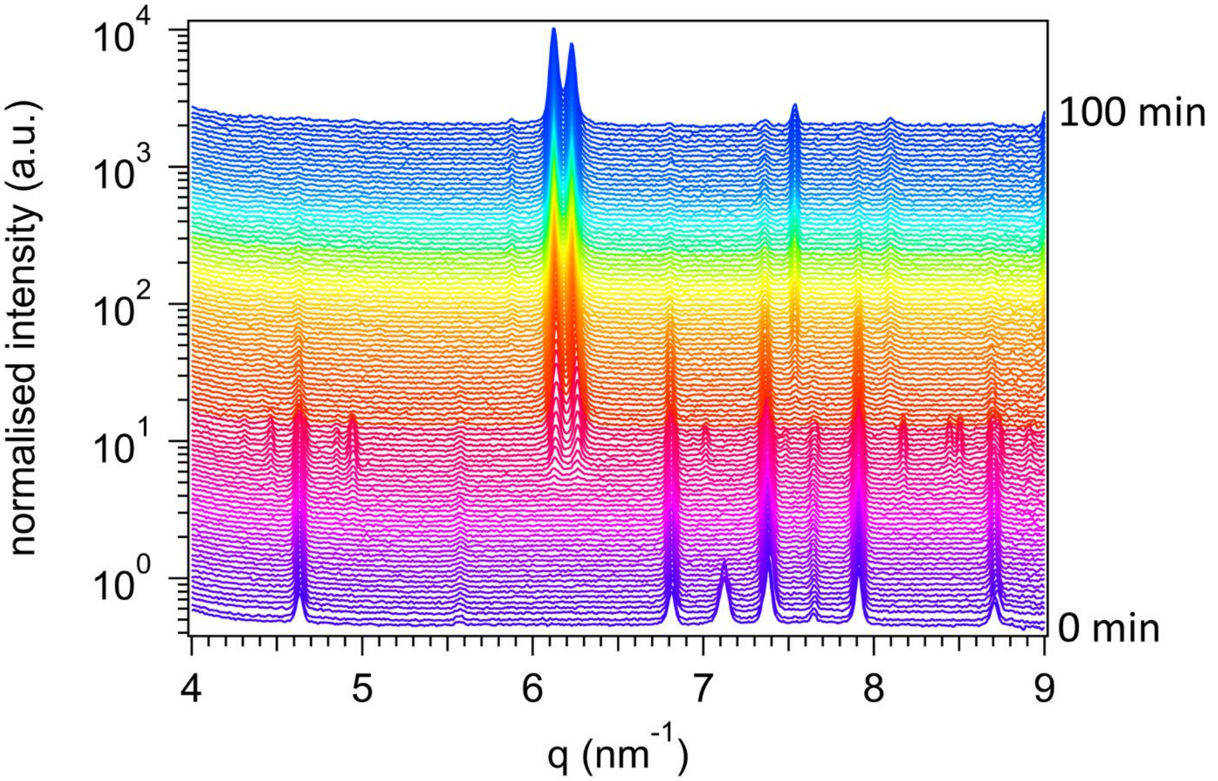

Figure S11: in-situ WAXS measurement of the crystallisation of 1-eq. CAT@M-BioHOF-1 with a flow of 1.1 sL/m of air with 100% RH and no flow of NH_3 vapour. The time between each frame is 1 min.

Kinetics of the crystallisation of M-BioHOF-1 and 1 eq.-CAT@M-BioHOF-1 calculated from the in-situ WAXS measurements with the vapour flow through set-up

Dependence on the humidity (RH%) of the crystallisation of M-BioHOF-1 and CAT@M-BioHOF-1.

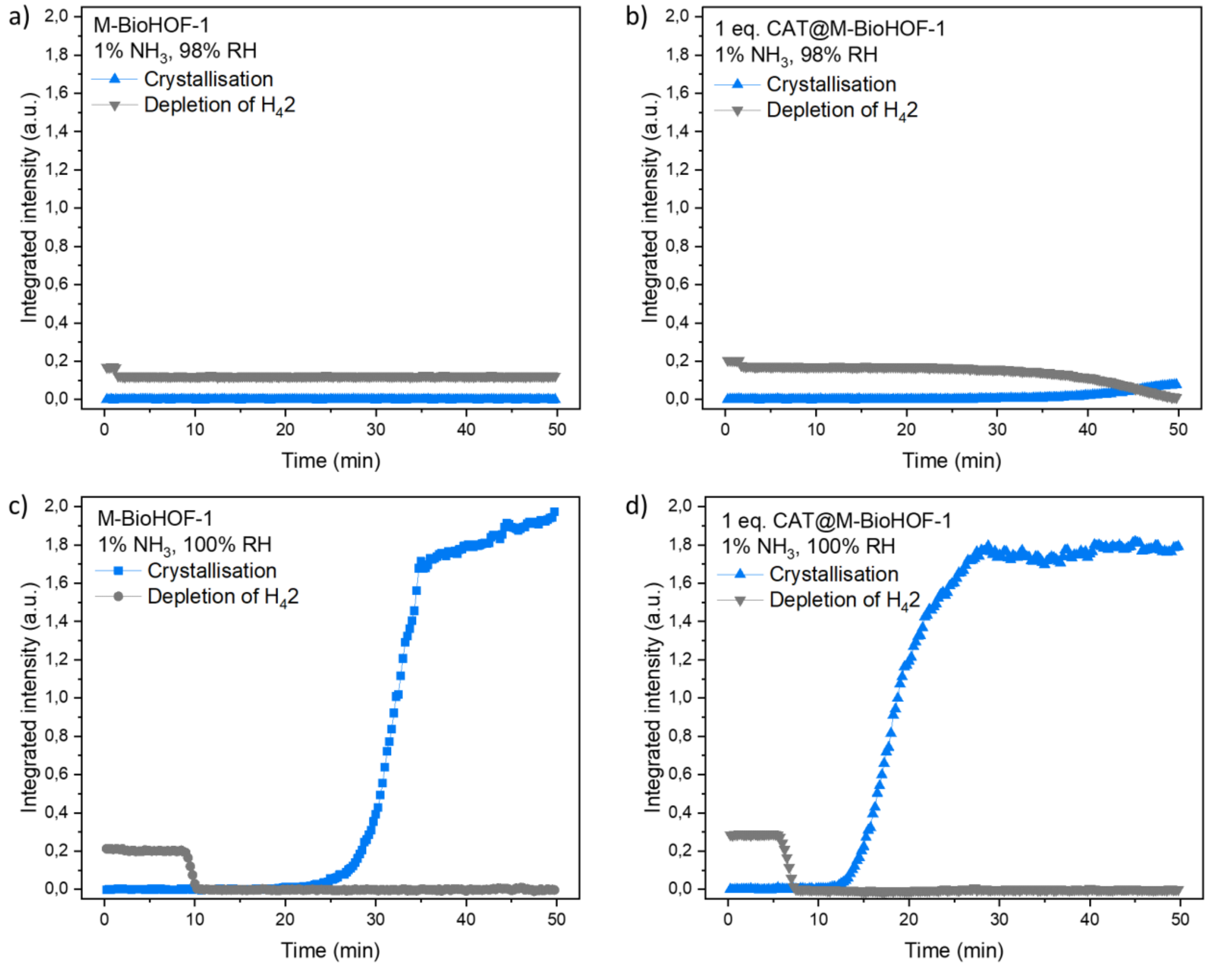

Figure S12: Time evolution of the normalized intensity integrated over the 6-6.45 nm^{-1} scattering vector (q) range (corresponding to the region of the (200) peak of BioHOF-1) and over the 7 to 7.2 nm^{-1} range (corresponding to one of the $H_4$2 precursor diffraction peak and representative of the depletion of the H42 precursor during the BioHOF-1 formation). Here, the influence of the humidity (RH: 98% or 100%) on the kinetics of crystallisation of M-BioHOF-1 (a and c, calculated respectively from the data showed in Fig. S16 and S17) and 1 eq. CAT@M-BioHOF-1 (b and d, calculated respectively from the data showed in Fig. S19 and S20) is shown.

Dependence on the concentration of NH_3 vapour (%NH_3) of the crystallisation of M-BioHOF-1

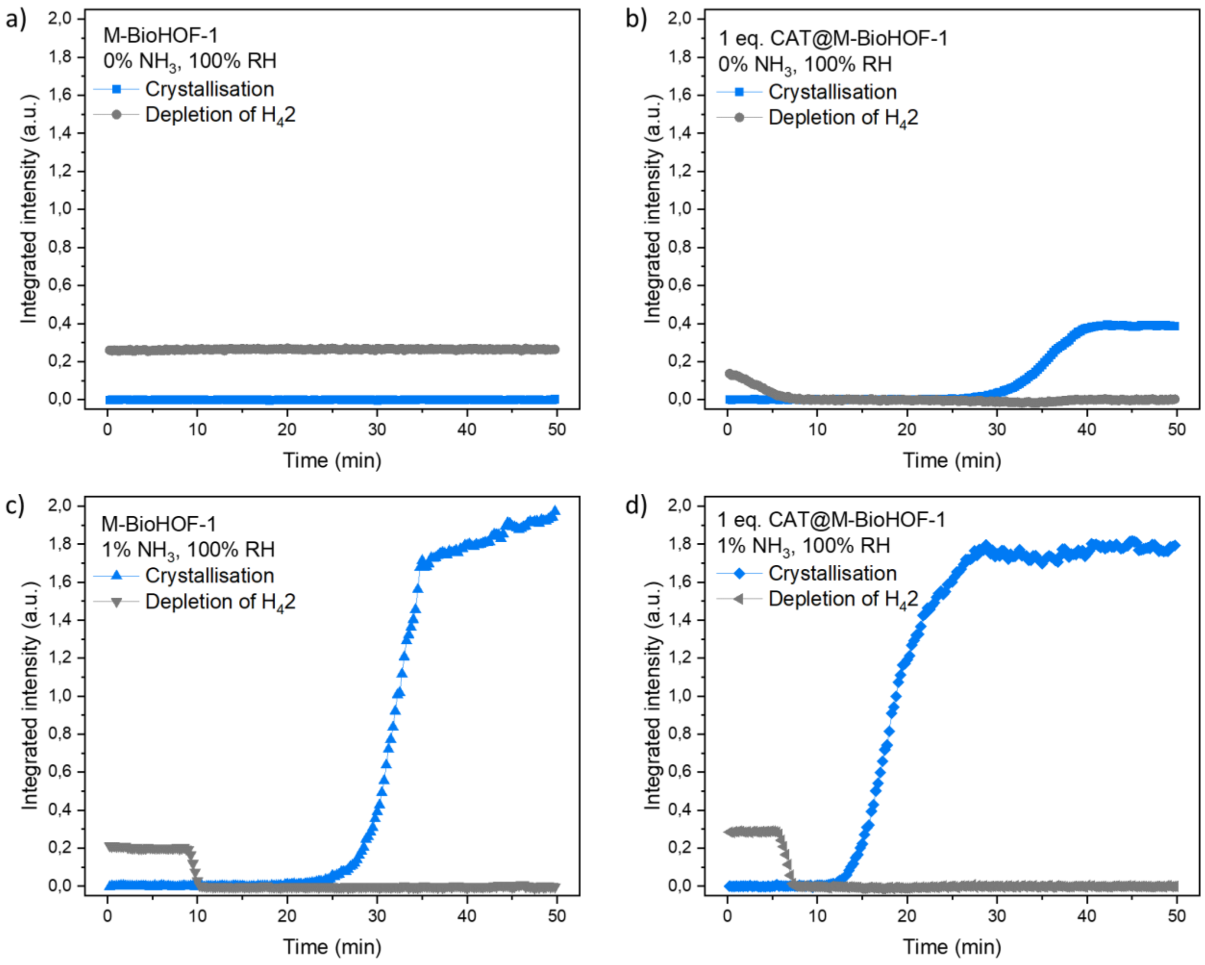

Figure S13: Time evolution of the normalized intensity integrated over the 6-6.45 nm^{-1} scattering vector (q) range (corresponding to the region of the (200) peak of BioHOF-1) and over the 7 to 7.2 nm^{-1} range (corresponding to one of the H_42 precursor diffraction peak and representative of the depletion of the H42 precursor during the BioHOF-1 formation). Here, the influence of the absence or presence of NH_3 (0% or 1% NH_3) on the kinetics of crystallisation of M-BioHOF-1 (a and c, calculated respectively from the data showed in Fig. S18 and S17) and 1 eq. CAT@M-BioHOF-1 (b and d, calculated respectively from the data showed in Fig. S21 and S20) is shown.

Dependence on the presence of CAT of the crystallisation of M-BioHOF-1

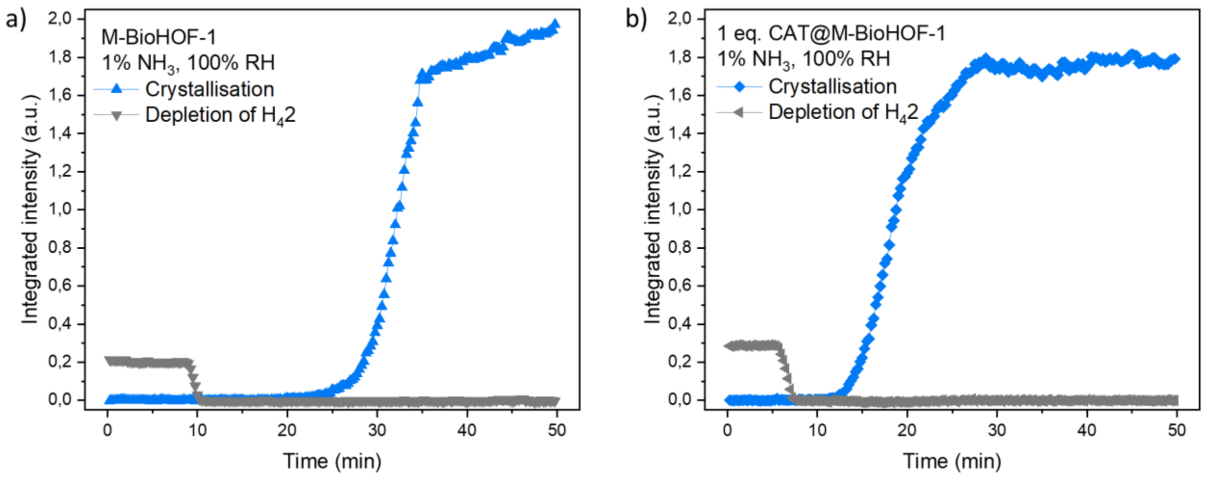

Figure S14: Time evolution of the normalized intensity integrated over the 6-6.45 nm^{-1} scattering vector (q) range (corresponding to the region of the (200) peak of BioHOF-1) and over the 7 to 7.2 nm^{-1} range (corresponding to one of the $H_4$2 precursor diffraction peak and representative of the depletion of the H42 precursor during the BioHOF-1 formation). Here, the influence of the absence or presence of CAT (0 or 1 eq.) on the kinetics of crystallisation of M-BioHOF-1 (a calculated from the data showed in Fig. S17) and 1 eq. CAT@M-BioHOF-1 (b, calculated from the data showed in Fig. S20) is shown.

Catalase enzymatic assays

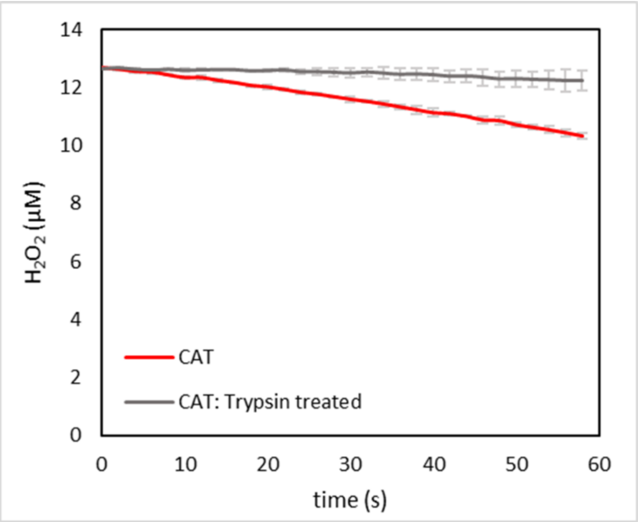

Figure S15: CAT enzymatic assays of the free CAT and the trypsin treated CAT with a CAT concentration of 0.025 mg/mL

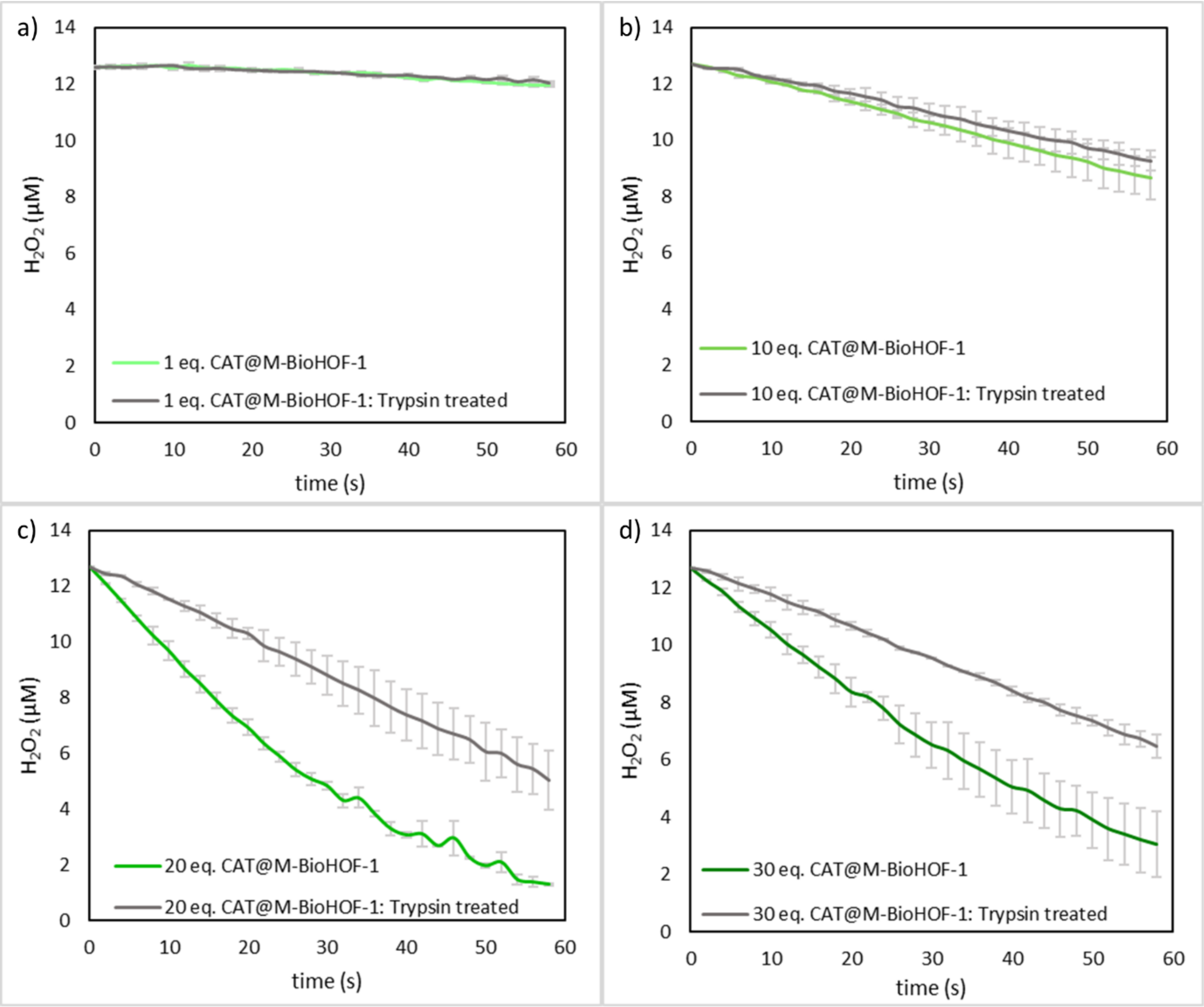

Figure S16: CAT enzymatic assays: a) 1 eq. CAT@M-BioHOF-1 and the trypsin treated 1 eq. CAT@M-BioHOF-1 sample with a biocomposite concentration of 1.29 mg/mL; b) 10 eq. CAT@M-BioHOF-1 and the trypsin treated 10 eq. CAT@M-BioHOF-1 sample with a biocomposite concentration of 1.65 mg/mL; c) 20 eq. CAT@M-BioHOF-1 and the trypsin treated 20 eq. CAT@M-BioHOF-1 sample with a biocomposite concentration of 2.16 mg/mL; d) 30 eq. CAT@M-BioHOF-1 and the trypsin treated 30 eq. CAT@M-BioHOF-1 sample with a biocomposite concentration of 1.17 mg/mL.

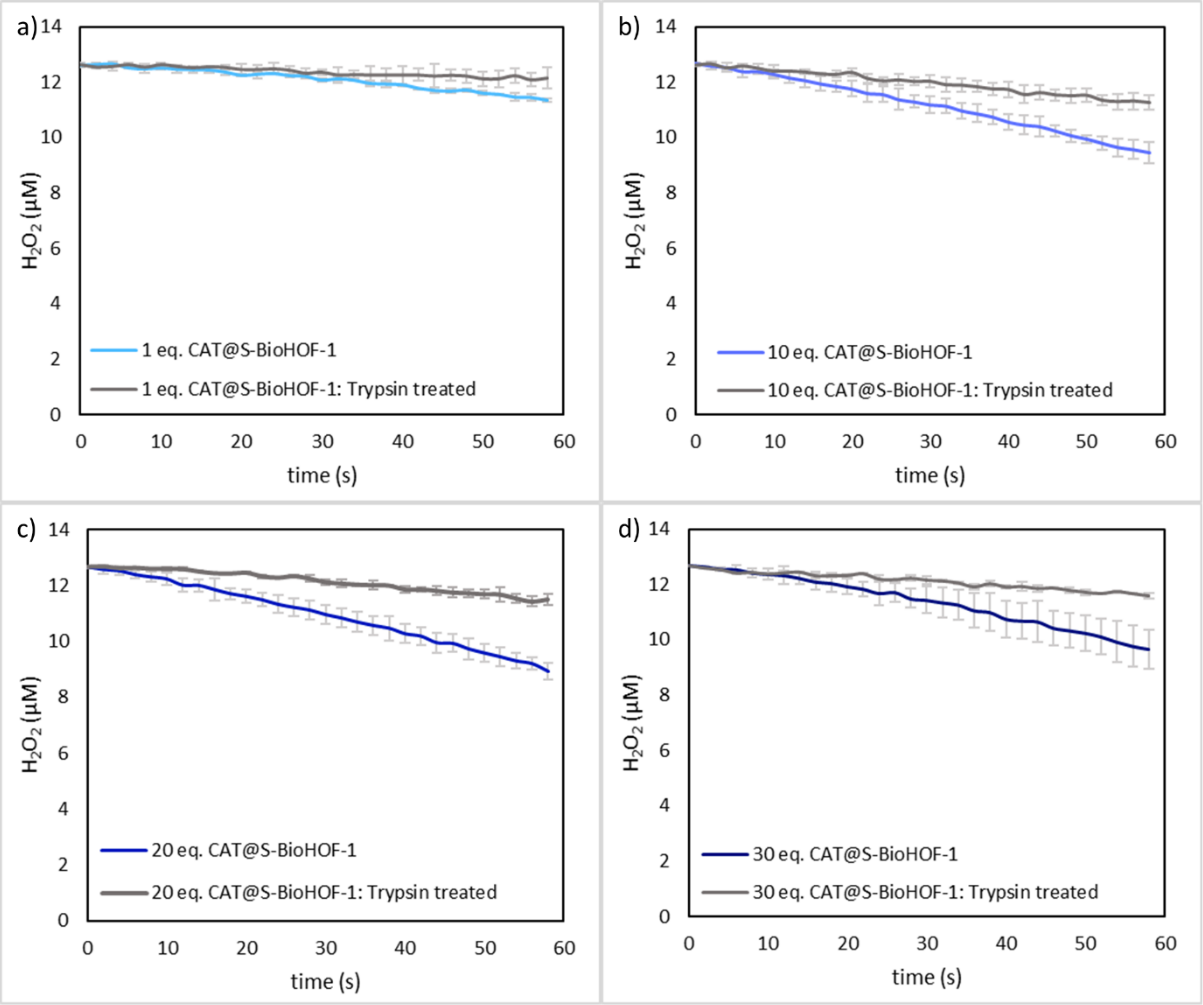

Figure S17: CAT enzymatic assays: a) 1 eq. CAT@S-BioHOF-1 and the trypsin treated 1 eq. CAT@S-BioHOF-1 sample with a biocomposite concentration of 0.51 mg/mL; b) 10 eq. CAT@S-BioHOF-1 and the trypsin treated 10 eq. CAT@S-BioHOF-1 sample with a biocomposite concentration of 0.81 mg/mL; c) 20 eq. CAT@S-BioHOF-1 and the trypsin treated 20 eq. CAT@S-BioHOF-1 sample with a biocomposite concentration of 0.87 mg/mL; d) 30 eq. CAT@S-BioHOF-1 and the trypsin treated 30 eq. CAT@S-BioHOF-1 sample with a biocomposite concentration of 0.91 mg/mL

Recyclability tests

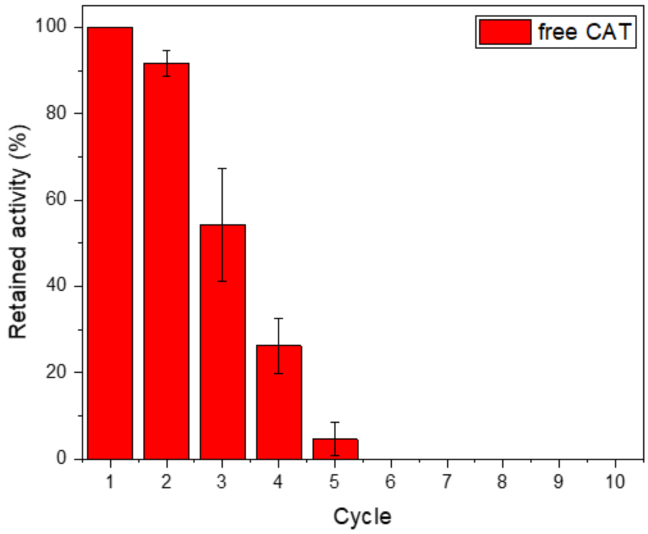

Figure S18: Relative activity of CAT after consecutive H_2O_2 (15 mM) decomposition cycles.

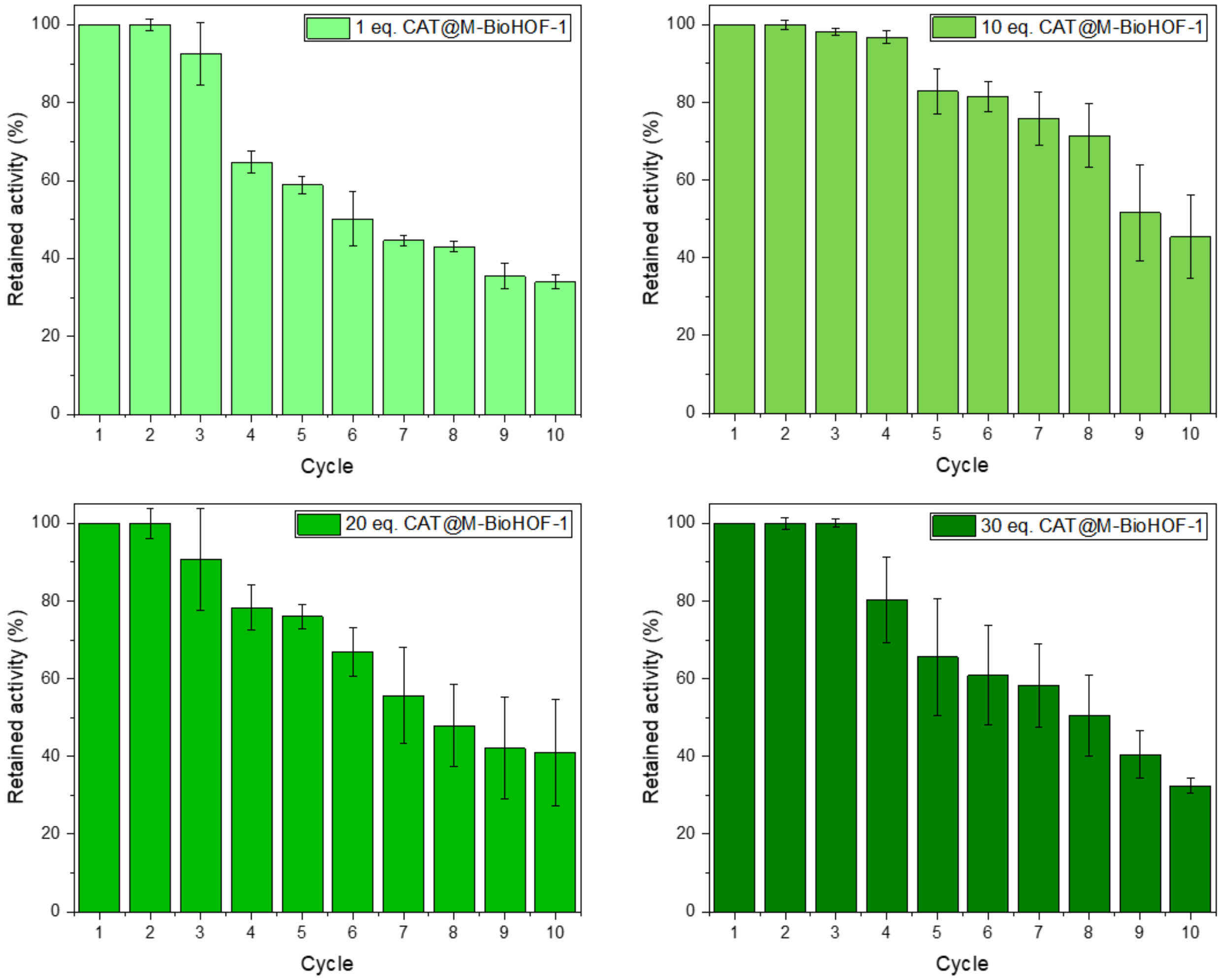

Figure S19: Relative activity of CAT@M-BioHOF-1 samples after consecutive H_2O_2 (15 mM) decomposition cycles.

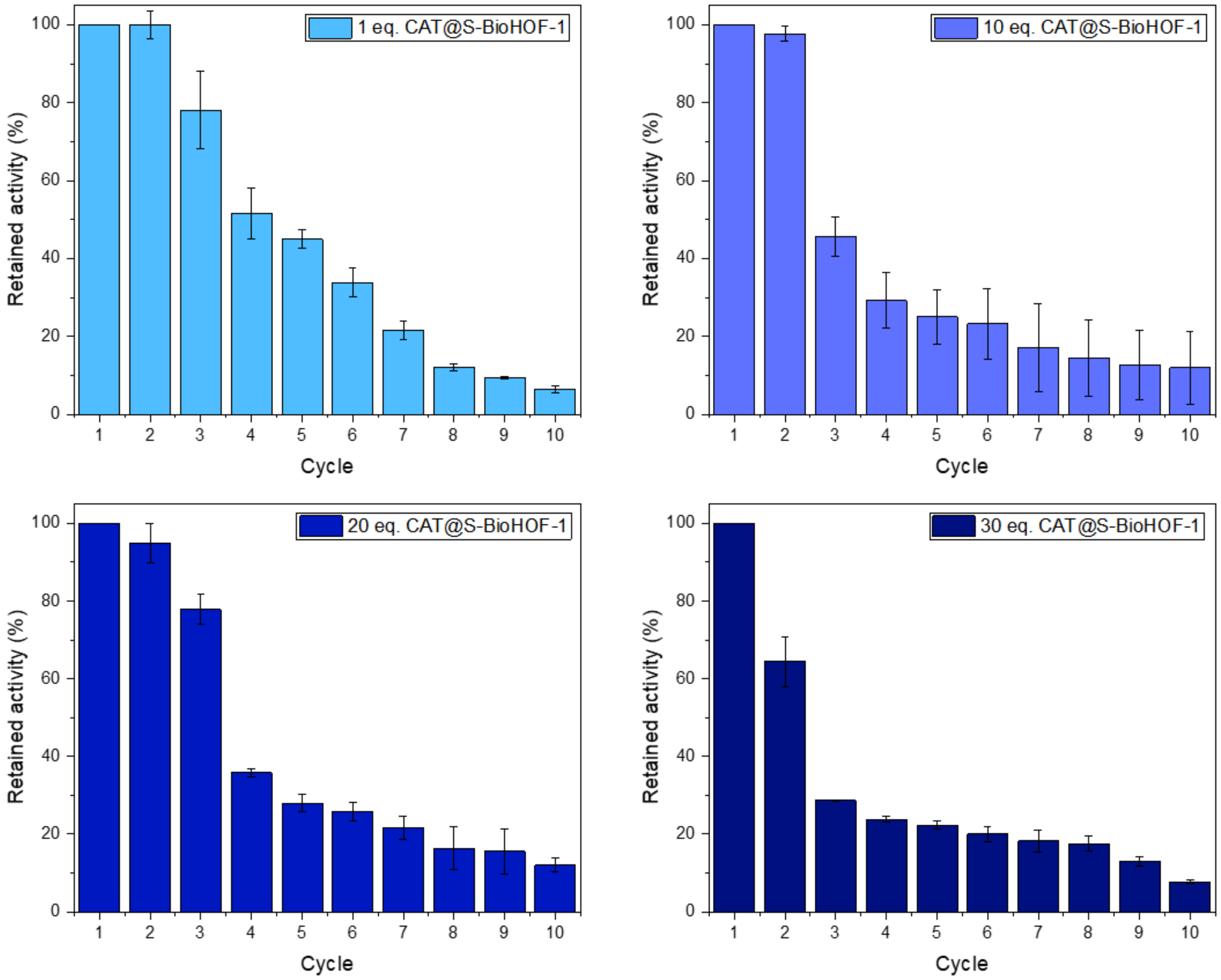

Figure S20: Relative activity of CAT@S-BioHOF-1 samples after consecutive H_2O_2 (15 mM) decomposition cycles.

CLSM

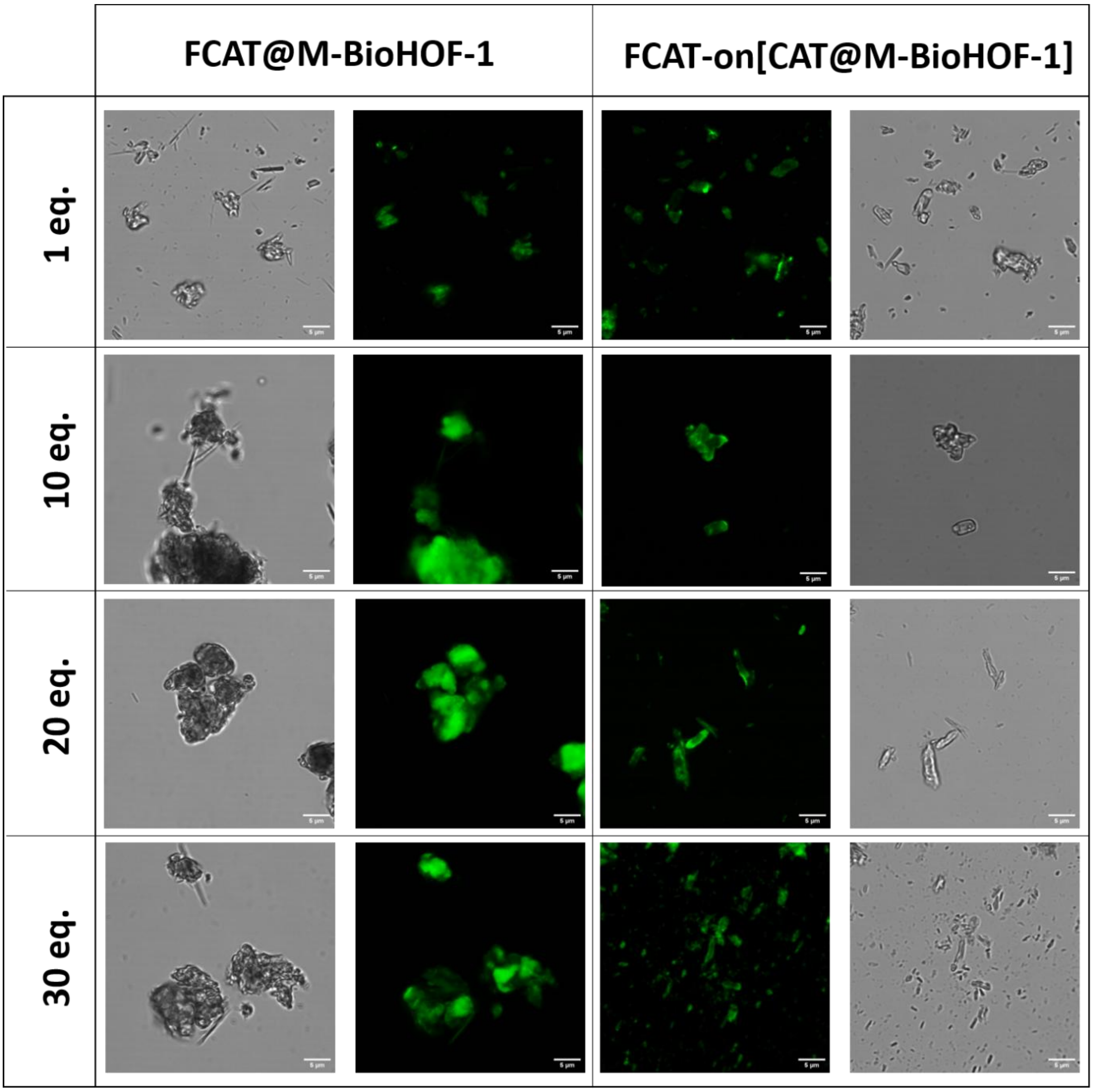

Figure S21: Bright field and confocal laser scanning microscopy (CLSM) images of FCAT@M-BioHOF-1 samples (left) prepared with 1, 10, 20 and 30 eq of FCAT. Bright field and confocal laser scanning microscopy (CLSM) images of FCAT-on[CAT@M-BioHOF-1] samples (right) prepared by adsorbing FCAT on the surface of CAT@M-BioHOF-1 samples synthetized with 1, 10, 20 and 30 eq of CAT.

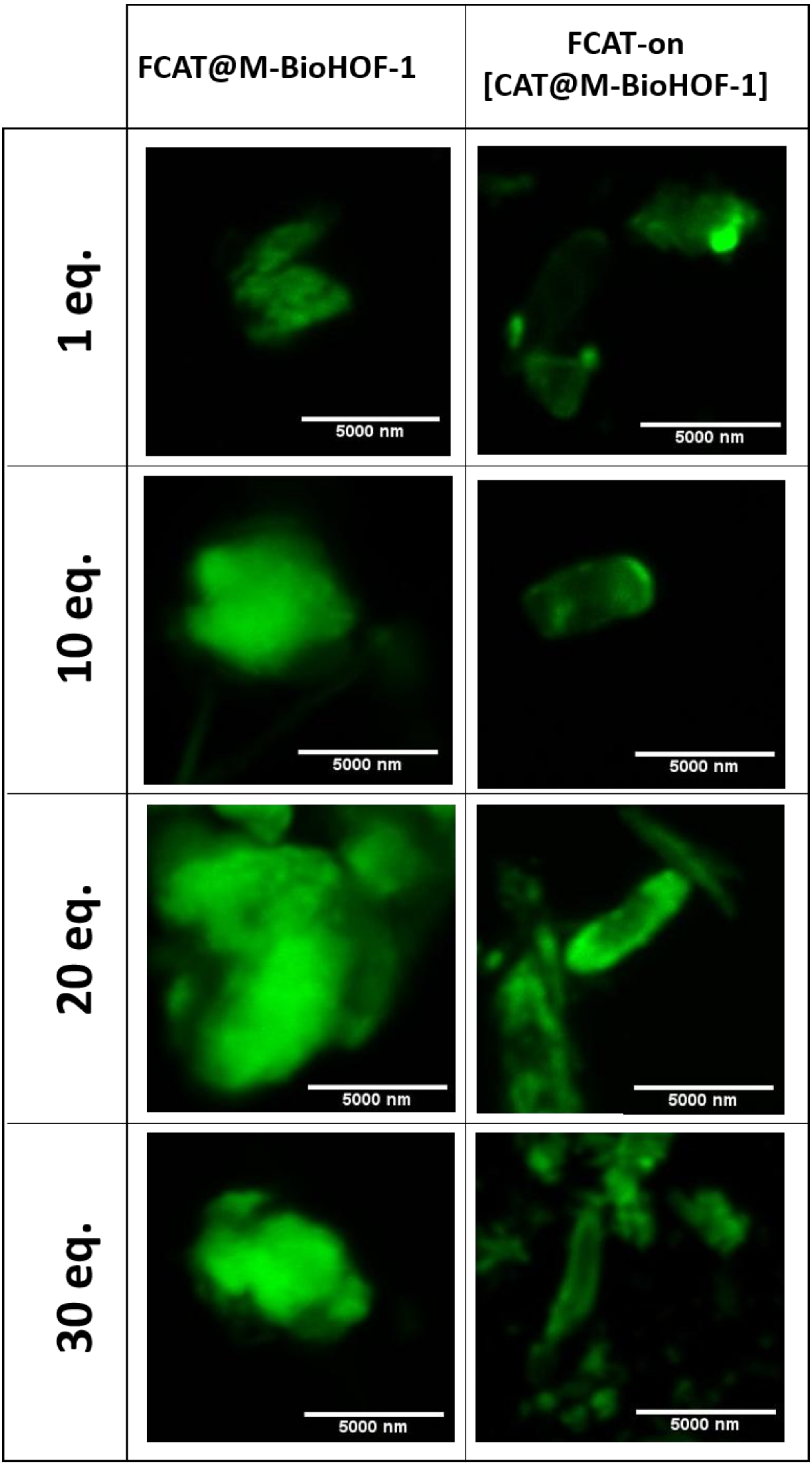

Figure S22: Digitally zoomed areas of the confocal laser scanning microscopy (CLSM) images from Figure S21 of FCAT@M-BioHOF-1 (left) and FCAT-on[CAT@M-BioHOF-1] samples (right).

XRD and IR characterisation of Ferritin@M-BioHOF-1 biocomposites

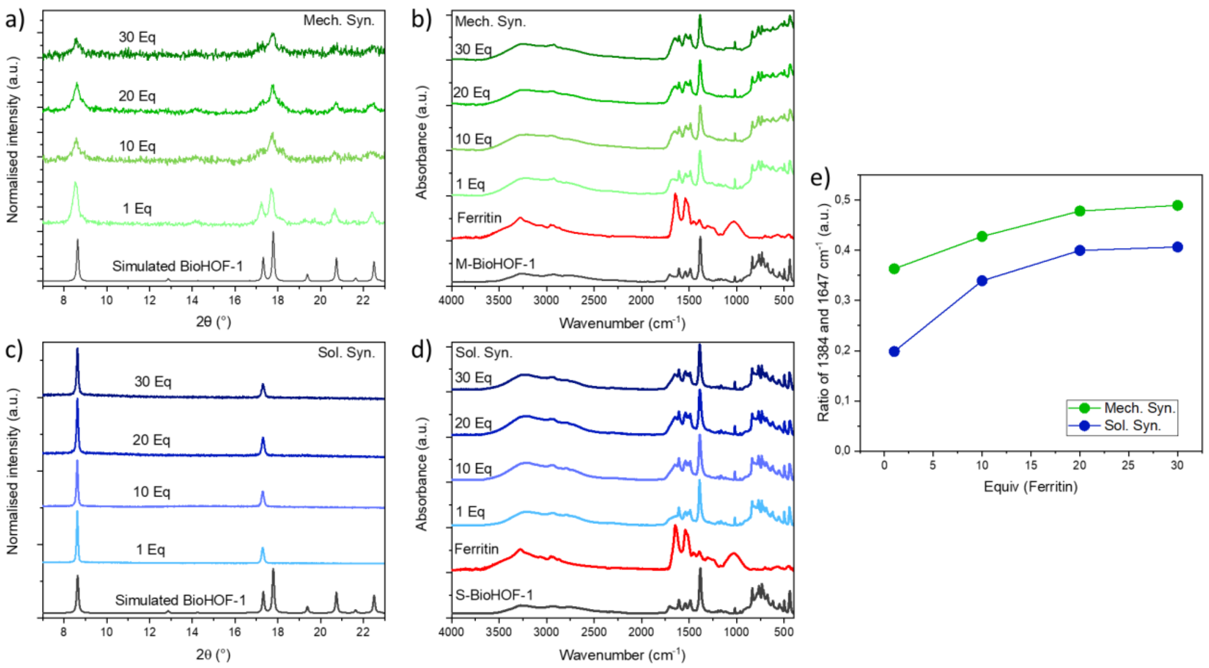

Figure S23: PXRD patterns (a) and ATR-IR spectra (b) of Ferritin@M-BioHOF-1 (Mech. Syn.). PXRD patterns (c) and ATR-IR spectra (d) of Ferritin@S-BioHOF-1 (Sol. Syn.). e) the ratio of the characteristic amide I band of Ferritin (1647 cm^{-1}) and the ip ring deform/sym carboxy stretch band of BioHOF-1 (1384 cm^{-1})[51] in the biocomposites.

Loading of Ferritin in Fer@M-BioHOF-1 and Fer@S-BioHOF-1 biocomposites

Table S3: Ferritin loading (wt%) calculated from ICP-OES.

Mechanochemical Synthesis	
**Ferritin equivalents**	**wt%**
1	11
10	24
20	50
30	45

Solvothermal Synthesis	
**Ferritin equivalents**	**wt%**
1	4
10	7
20	9
30	10

TEM micrographs of the Ferritin@M-BioHOF-1 biocomposites

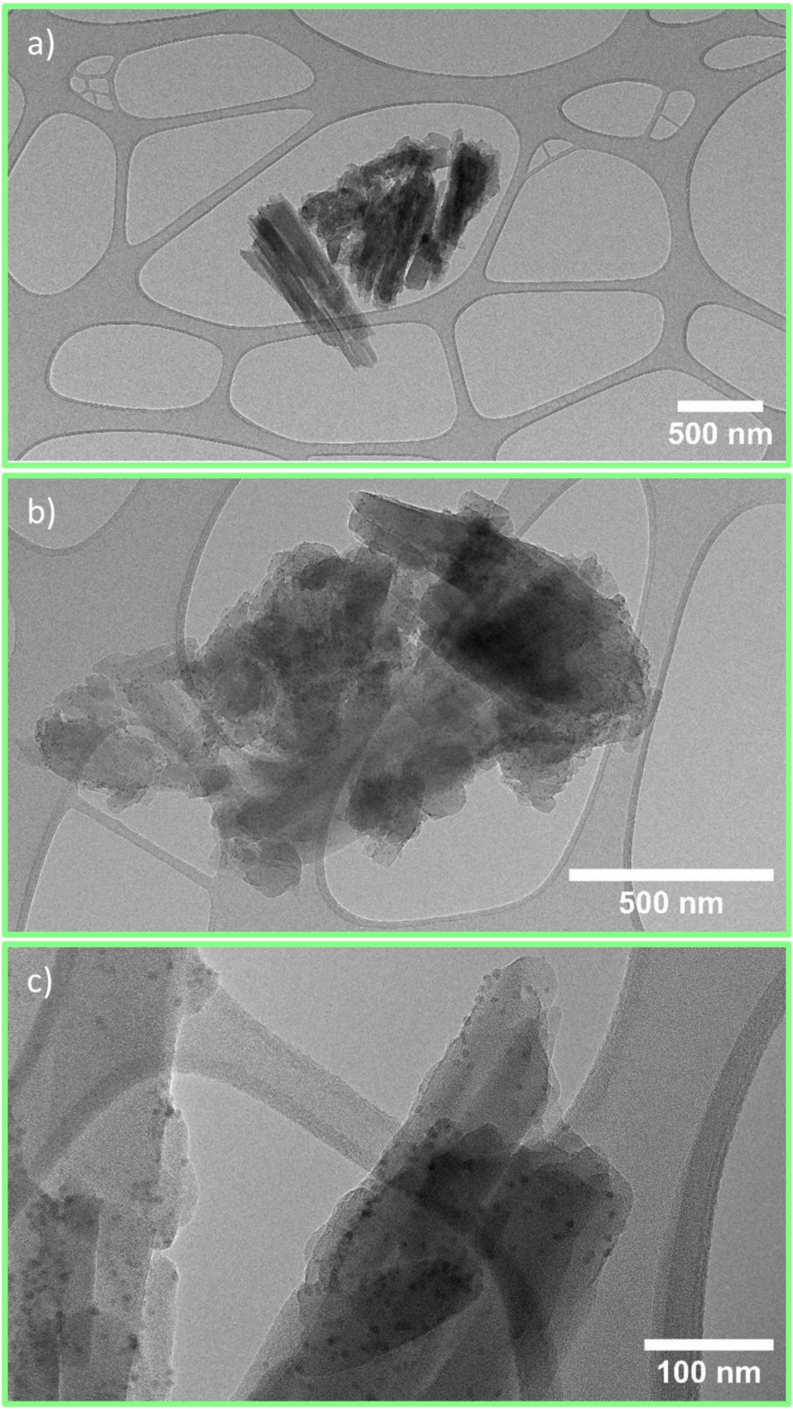

Figure S24: TEM micrographs of 1-eq. Ferritin@M-BioHOF-1: a) magnification of 11.5 kx, b) magnification of 27.5 kx and c) magnification of 88 kx.

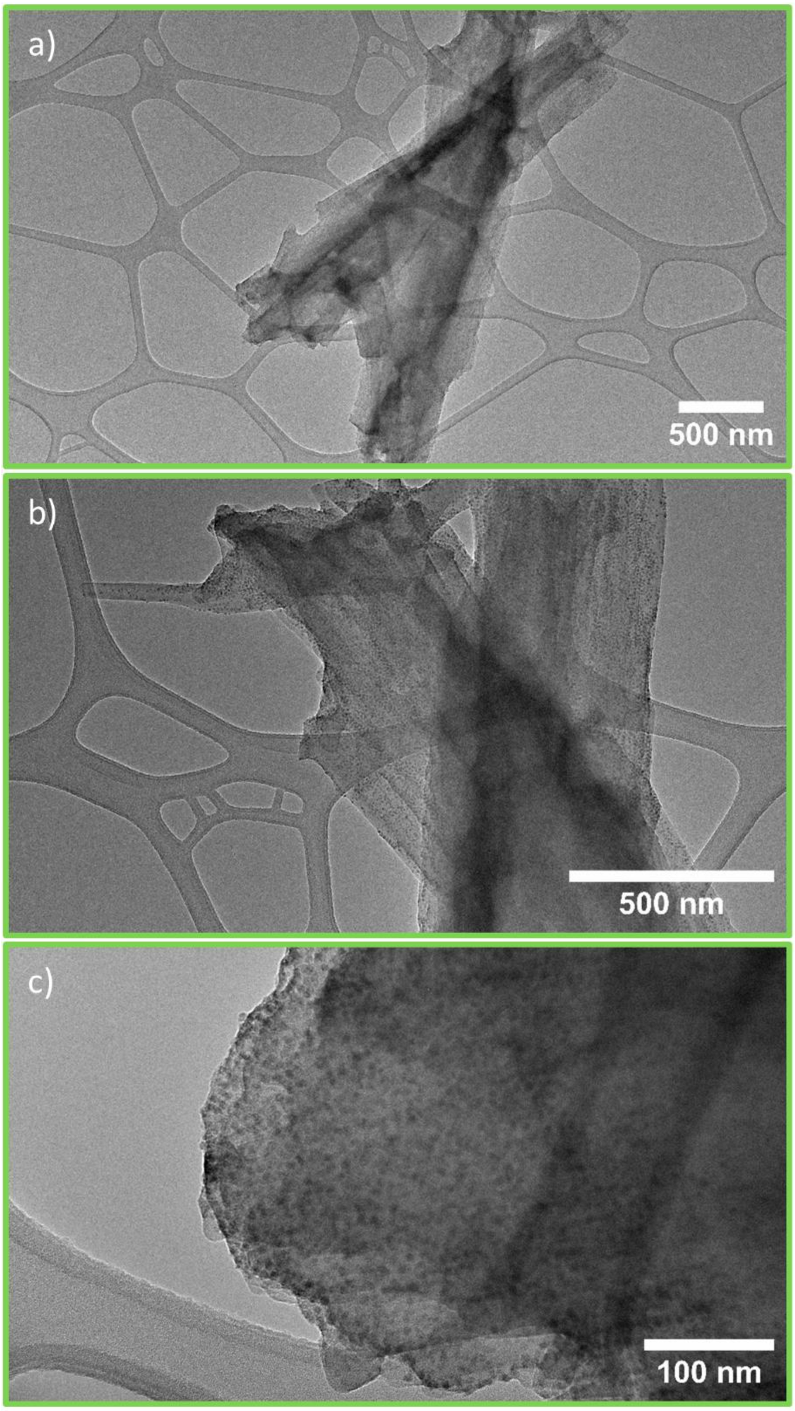

Figure S25: TEM micrographs of 10-eq. Ferritin@M-BioHOF-1: a) magnification of 11.5 kx, b) magnification of 27.5 kx and c) magnification of 88 kx.

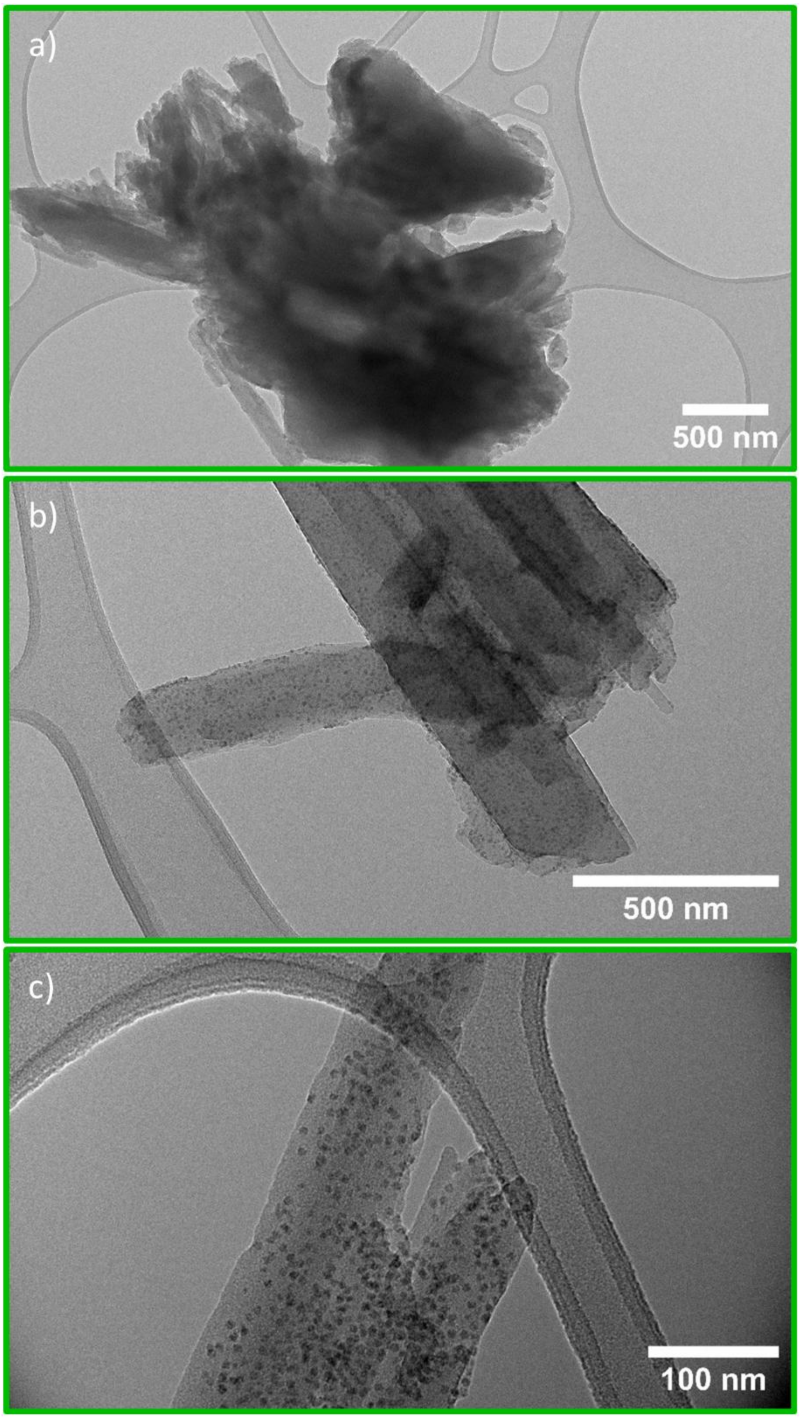

Figure S26: TEM micrographs of 20-eq. Ferritin@M-BioHOF-1: a) magnification of 11.5 kx, b) magnification of 27.5 kx and c) magnification of 88 kx.

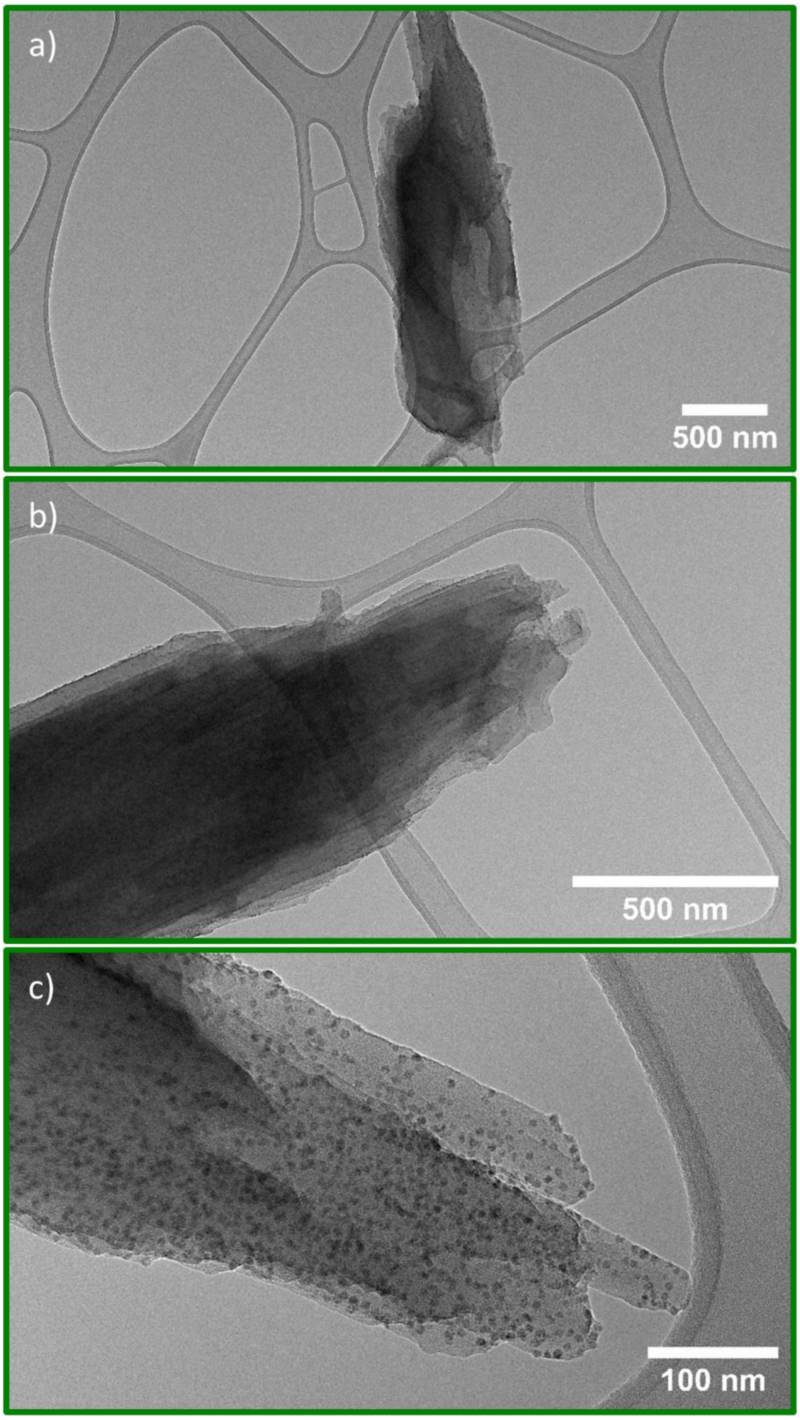

Figure S27: TEM micrographs of 30-eq. Ferritin@M-BioHOF-1: a) magnification of 11.5 kx, b) magnification of 27.5 kx and c) magnification of 88 kx.

TEM micrographs of the Ferritin@S-BioHOF-1 biocomposites

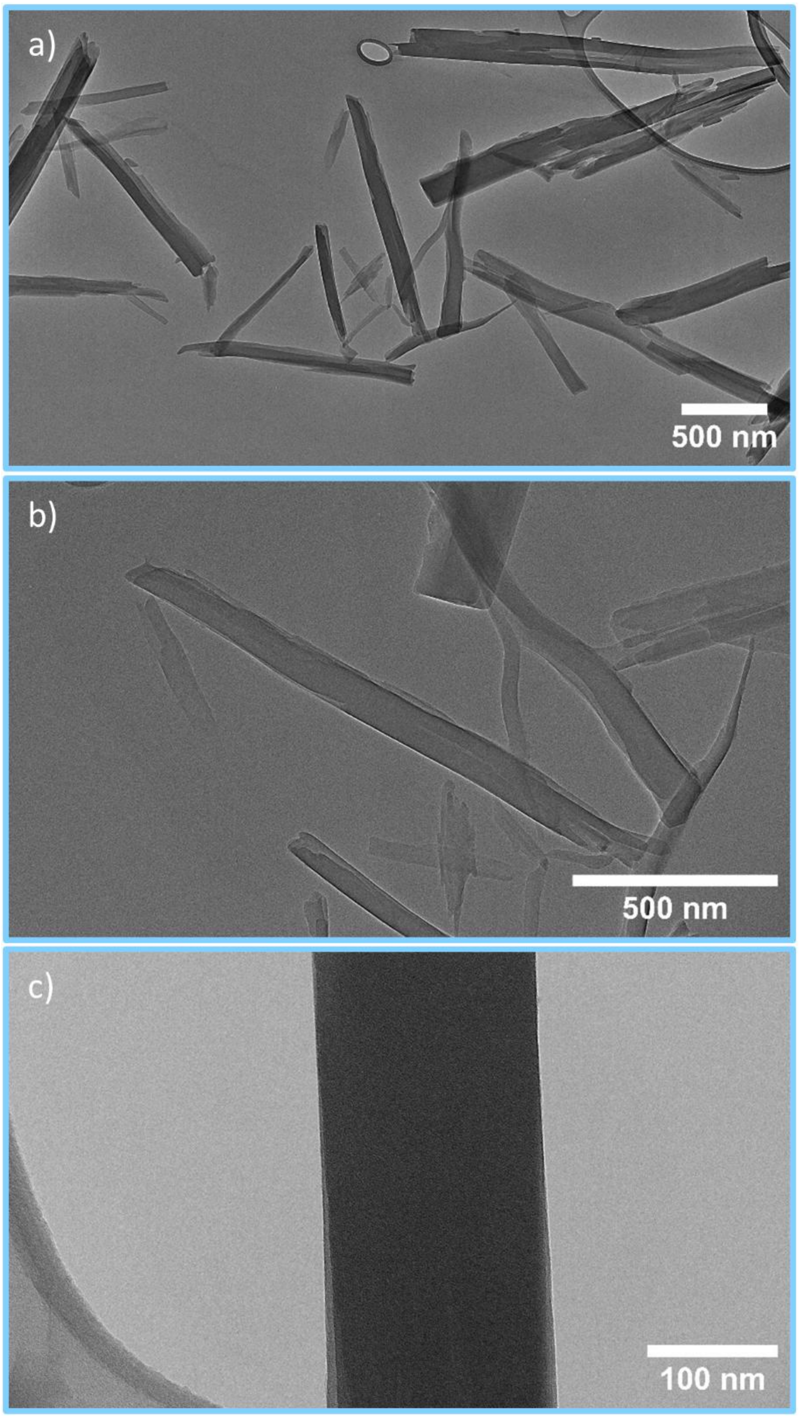

Figure S28: TEM micrographs of 0-eq. Ferritin@S-BioHOF-1: a) magnification of 11.5 kx, b) magnification of 27.5 kx and c) magnification of 88 kx.

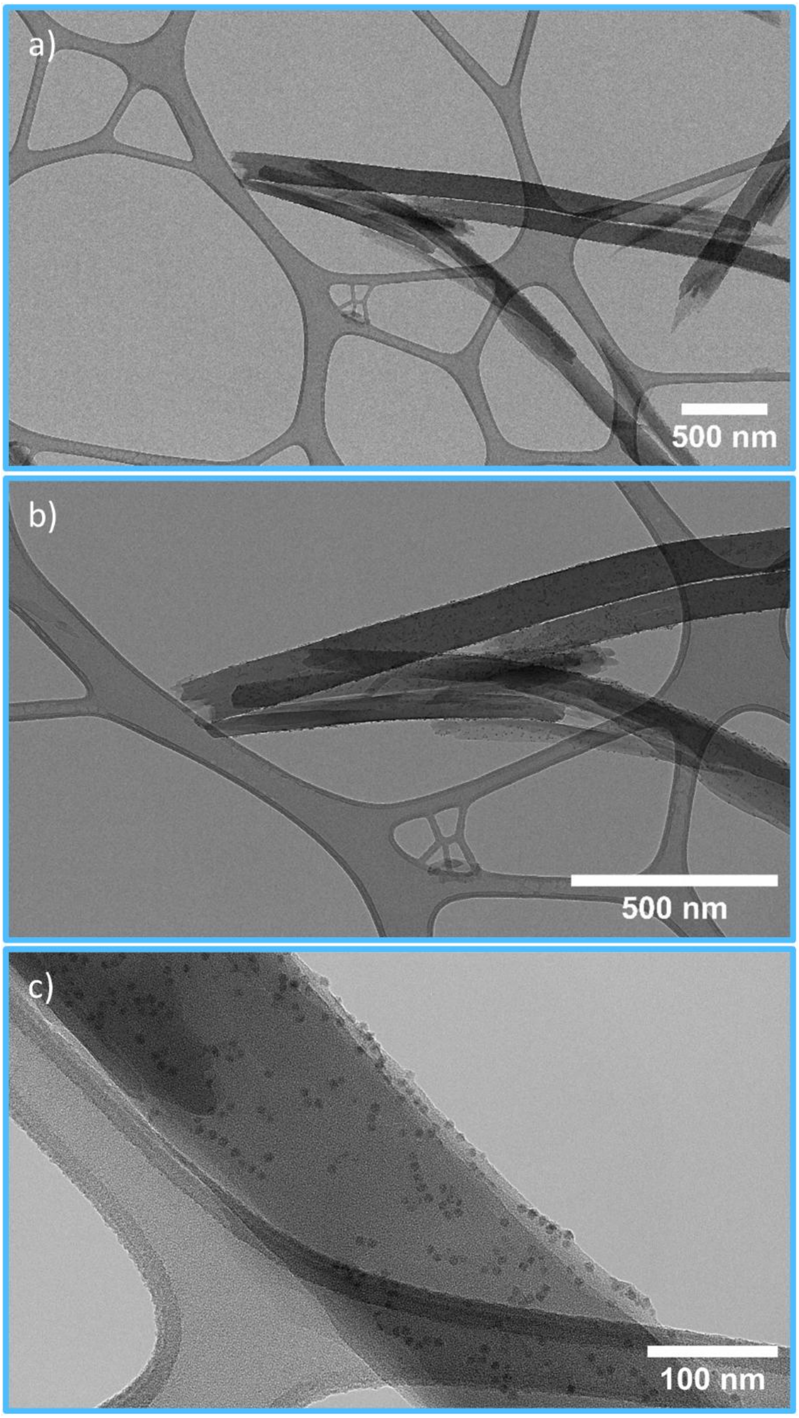

Figure S29: TEM micrographs of 1-eq. Ferritin@S-BioHOF-1: a) magnification of 11.5 kx, b) magnification of 27.5 kx and c) magnification of 88 kx.

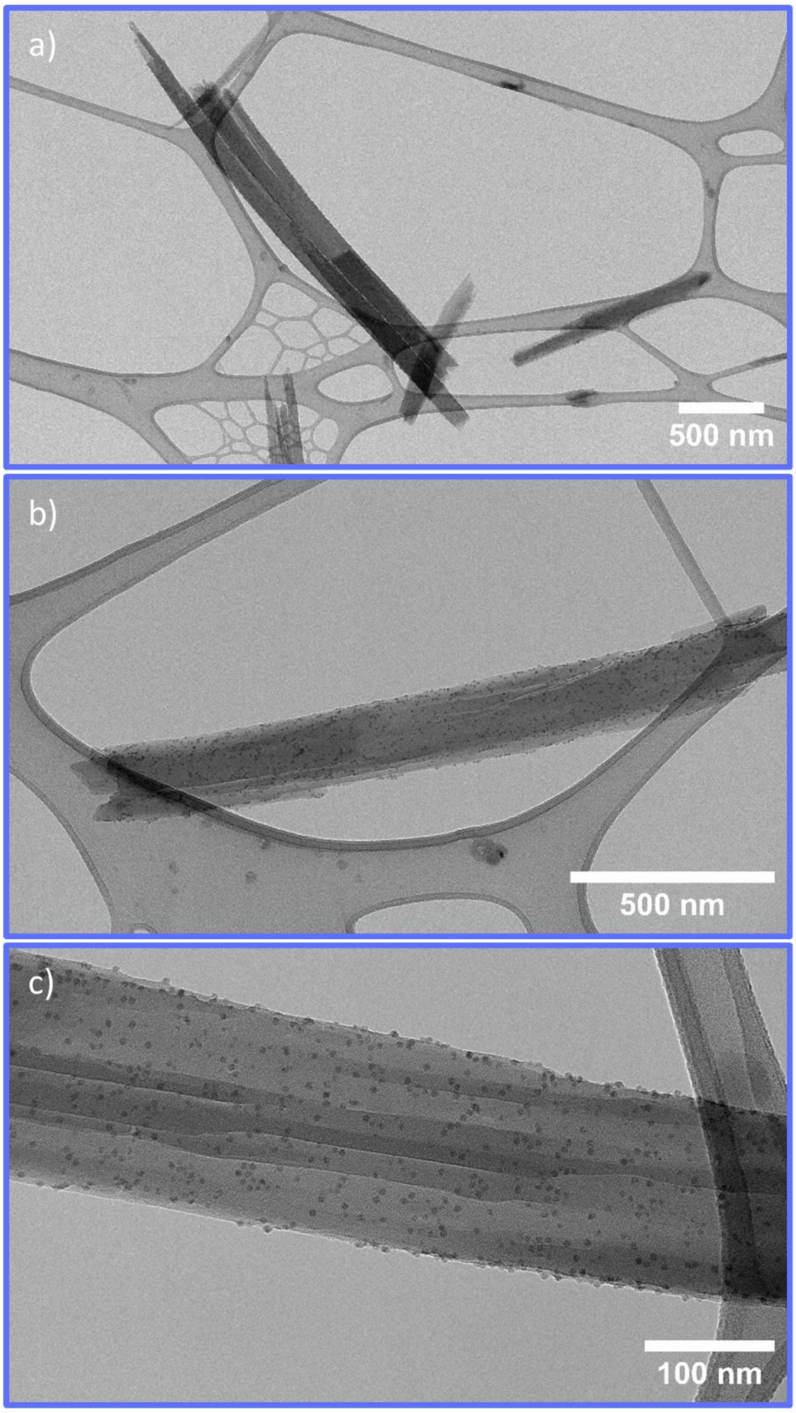

Figure S30: TEM micrographs of 10-eq. Ferritin@S-BioHOF-1: a) magnification of 11.5 kx, b) magnification of 27.5 kx and c) magnification of 88 kx.

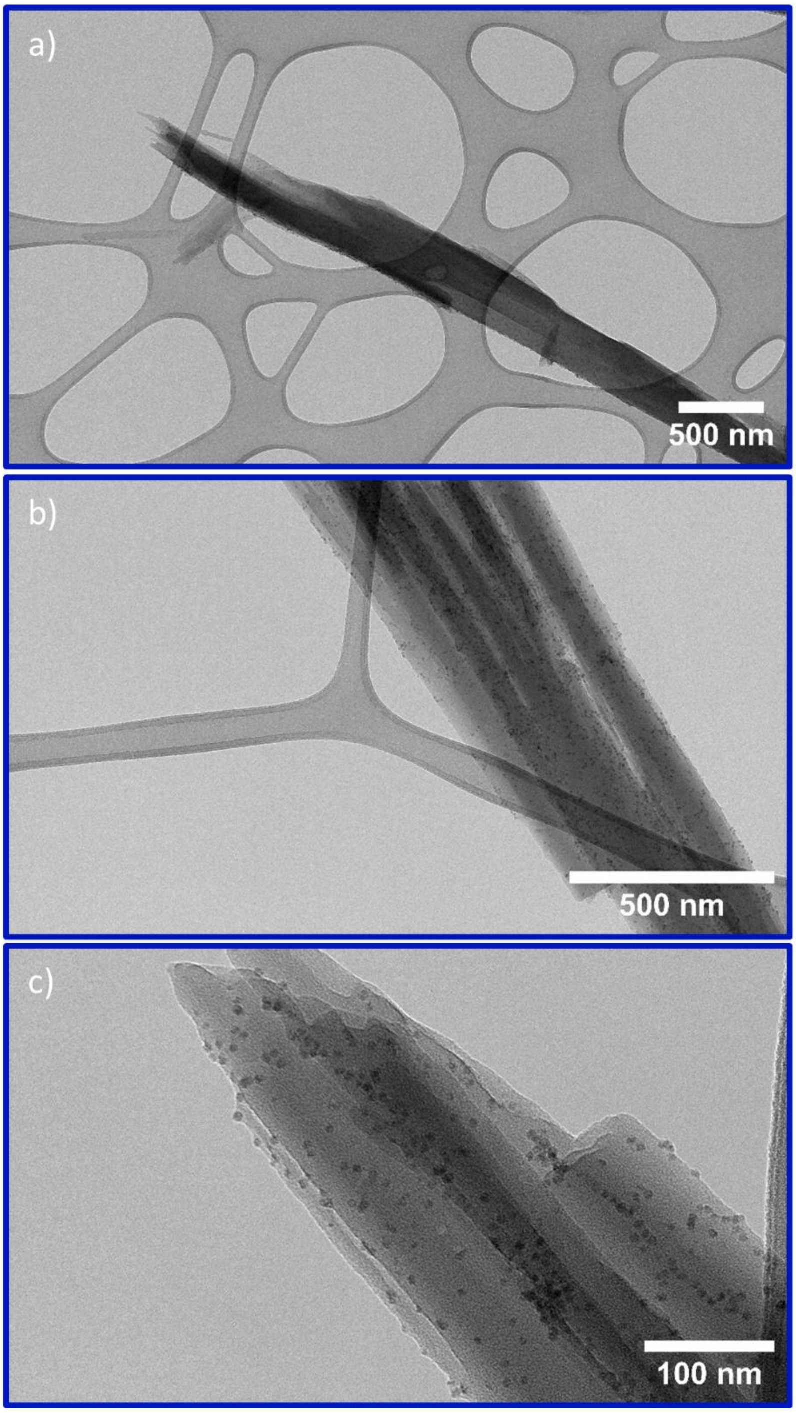

Figure S31: TEM micrographs of 20-eq. Ferritin@S-BioHOF-1: a) magnification of 11.5 kx, b) magnification of 27.5 kx and c) magnification of 88 kx.

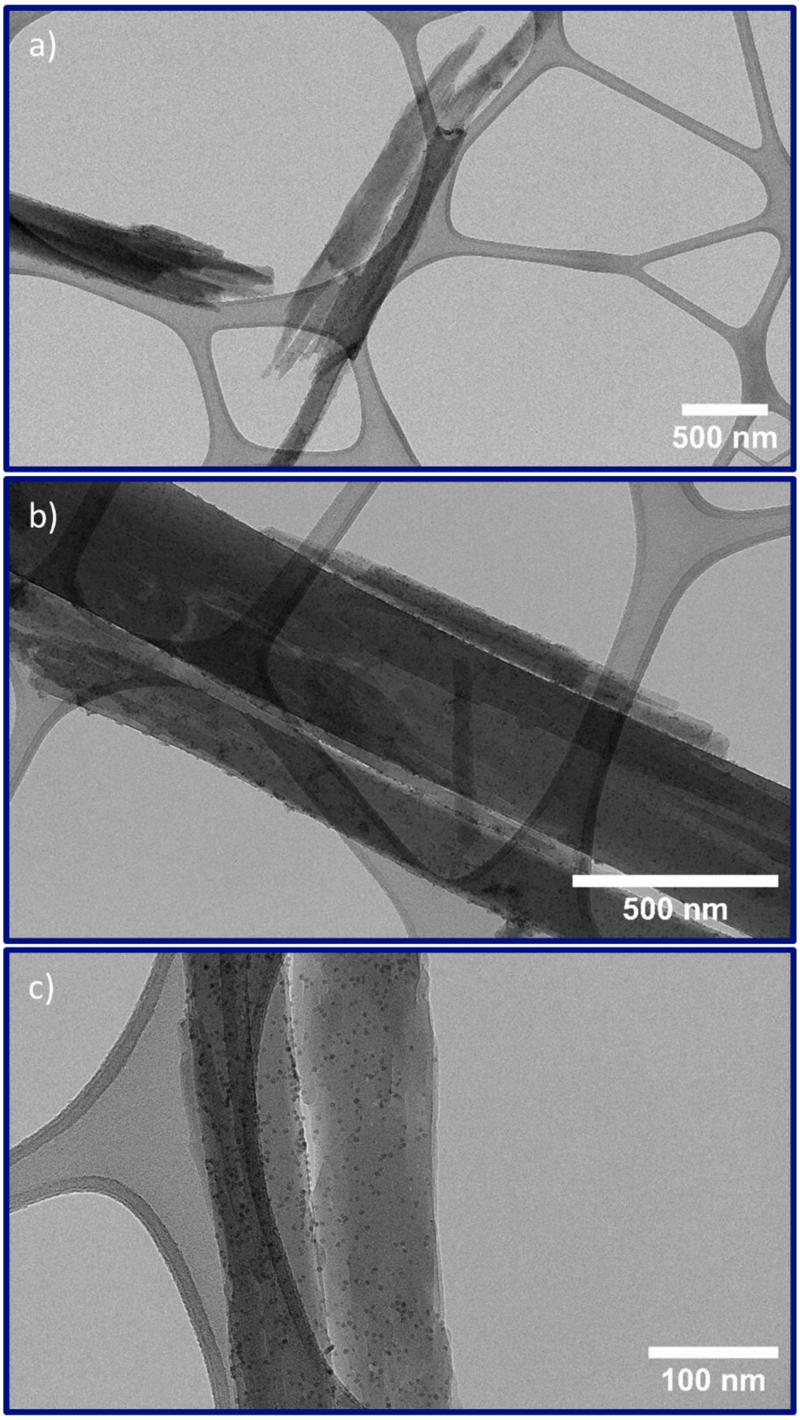

Figure S32: TEM micrographs of 30-eq. Ferritin@S-BioHOF-1: a) magnification of 11.5 kx, b) magnification of 27.5 kx and c) magnification of 88 kx.

References

[13] W. Liang, F. Carraro, M. B. Solomon, S. G. Bell, H. Amenitsch, C. J. Sumby, N. G. White, P. Falcaro, C. J. Doonan, *J. Am. Chem. Soc.* **2019**, *141*, 14298–14305.

[57] H. Amenitsch, M. Rappolt, M. Kriechbaum, H. Mio, P. Laggner, S. Bernstorff, *J Synchrotron Rad* **1998**, *5*, 506–508.

[58] B. Marmiroli, B. Sartori, A. R. Kyvik, I. Ratera, H. Amenitsch, *Front. Mater.* **2021**, *8*, 686353.

[51] C. Ennis, D. R. T. Appadoo, S. A. Boer, N. G. White, *Phys. Chem. Chem. Phys.* **2022**, *24*, 10784–10797.

[59] E. P. Perman, *J. Chem. Soc., Trans.* **1903**, *83*, 1168–1184.
